# HupB, a nucleoid-associated protein, is critical for survival of *Mycobacterium tuberculosis* under host-mediated stresses and for enhanced tolerance to key first-line antibiotics

**DOI:** 10.3389/fmicb.2022.937970

**Published:** 2022-08-22

**Authors:** Niti Singh, Nishant Sharma, Padam Singh, Manitosh Pandey, Mohd Ilyas, Lovely Sisodiya, Tejaswini Choudhury, Tannu Priya Gosain, Ramandeep Singh, Krishnamohan Atmakuri

**Affiliations:** ^1^Infection and Immunology Group, Translational Health Science and Technology Institute, Faridabad, Haryana, India; ^2^Manipal University, Manipal, Karnataka, India; ^3^Department of Life Sciences, ITM University, Gwalior, Madhya Pradesh, India; ^4^School of Life Sciences, Jawaharlal Nehru University, New Delhi, India

**Keywords:** HupB, Hlp, stress, antibiotics, SD1, *Mycobacterium tuberculosis*, *Mycobacterium smegmatis*

## Abstract

To survive and establish its niche, *Mycobacterium tuberculosis* (Mtb) engages in a steady battle against an array of host defenses and a barrage of antibiotics. Here, we demonstrate that Mtb employs HupB, a nucleoid-associated protein (NAP) as its key player to simultaneously battle and survive in these two stress-inducing fronts. Typically, NAPs are key to bacterial survival under a wide array of environmental or host-mediated stresses. Here, we report that for Mtb to survive under different macrophage-induced assaults including acidic pH, nutrient depletion, oxidative and nitrosative stresses, HupB presence is critical. As expected, the *hupB* knockout mutant is highly sensitive to these host-mediated stresses. Furthermore, Mtb aptly modulates HupB protein levels to overcome these stresses. We also report that HupB aids Mtb to gain tolerance to high levels of rifampicin (RIF) and isoniazid (INH) exposure. Loss of *hupB* makes Mtb highly susceptible to even short exposures to reduced amounts of RIF and INH. Overexpressing *hupB* in Mtb or complementing *hupB* in the *hupB* knockout mutant triggers enhanced survival of Mtb under these stresses. We also find that upon loss of *hupB*, Mtb significantly enhances the permeability of its cell wall by modulating the levels of several surface lipids including phthiocerol dimycocerosates (PDIMs), thus possibly influencing overall susceptibility to host-mediated stresses. Loss of *hupB* also downregulates efflux pump expression possibly influencing increased susceptibility to INH and RIF. Finally, we find that therapeutic targeting of HupB with SD1, a known small molecule inhibitor, significantly enhances Mtb susceptibility to INH and THP-1 macrophages and significantly reduces MIC to INH. Thus, our data strongly indicate that HupB is a highly promising therapeutic target especially for potential combinatorial shortened therapy with reduced INH and RIF doses.

## Introduction

Annually, worldwide, tuberculosis (TB) causes ~10 million fresh individuals to fall sick and kills at least a million individuals (Global tuberculosis report, 2020). Majority of this burden is concentrated in low- and middle-income countries where TB remains endemic. TB-triggered mortality has continued despite diligent BCG vaccination and easy access to first-line anti-TB drugs (Global tuberculosis report, 2020). With rapid emergence of multi- and extremely drug resistant strains of *Mycobacterium tuberculosis* (Mtb) and their increasing contribution to TB burden, second-line anti-TB drugs have also become readily available (Seung et al., 2015; Prasad et al., 2017). However, with treatment duration still lasting for 6–24 months, most patients experience one or more toxicity issues including liver damage, gastritis, vomiting, heartburn, reduced appetite, visual impairment, and rashes (Yee et al., 2003; Forget and Menzies, 2006; Castro et al., 2015; Madhav et al., 2015; Prasad et al., 2019). Consequently, there is widespread poor compliance to anti-TB therapy.

Thus, there is an urgent need to identify novel drug molecules against new pathogen-specific targets that not only exhibit superior efficacy but also significantly shorten treatment regimens, reduce total drug intake, and exhibit minimal to no side effects (Duncan and Barry, 2004; Singh and Mizrahi, 2017; Wellington and Hung, 2018). Since anti-TB therapy is primarily combinatorial, any new identified drug molecule must also be able to work seamlessly with the existing first-line drugs. If such a molecule can show an additive or synergistic effect with the existing first-line drugs and simultaneously reduce the dosage of the other drugs (in combination), it holds a huge promise as a potential novel drug (Duncan and Barry, 2004; Singh and Mizrahi, 2017; Wellington and Hung, 2018; Shetye et al., 2020).

Consequently, several Mtb proteins including nucleoid-associated histone-like proteins (NAPs) are under evaluation as novel therapeutic targets (Pinault et al., 2013; Bhowmick et al., 2014; Peraman et al., 2021). Typically, in bacteria, NAPs are known to orchestrate nucleoid dynamics, operate as global transcription modulators, shield nucleoid under stress conditions, and alter cellular responses to stress (Hołówka and Zakrzewska-Czerwińska, 2020). They are small positively charged transcriptional gene regulators that modulate DNA-dependent processes to influence local and/or global gene expression landscapes (Dorman and Deighan, 2003; Dillon and Dorman, 2010; Priyadarshini et al., 2013; Kriel et al., 2018; Datta et al., 2019). Mycobacterial NAPs are no exception. Mtb encodes at least six different NAPs, *viz*., EspR, HupB (also referred to as MtbHU/HU), Lsr2, mIHF, MDP2, and NapM (Kriel et al., 2018). Lsr2 and EspR are DNA-bridging proteins, with the former influencing cell wall biosynthesis, oxidative stress, and antibiotic resistance (Chen et al., 2008; Kriel et al., 2018) and the latter regulating secretion of virulent proteins by ESX-1 through two 2-component regulatory systems, viz., PhoP-PhoR and MprA-MprB (Cao et al., 2015; Anil Kumar et al., 2016). In contrast, MDP2, mIHF, and NapM directly bind curved DNA, promote non-curved DNA compactions, and influence Mtb growth *in vitro* (Kriel et al., 2018).

HupB is an Mtb-encoded HU-like protein of *E. coli* and a prominent member of Mtb-encoded NAPs (Kriel et al., 2018; Datta et al., 2019). Not surprisingly, HupB promotes nucleoid compaction by associating with ds DNA (Bhowmick et al., 2014; Gupta et al., 2014; Hołówka et al., 2017). It is also implicated in several important biological functions including (i) immune-modulation (Prabhakar et al., 1998), (ii) adhesion to alveolar macrophages (Pandey et al., 2014a; Kalra et al., 2018; Yaseen et al., 2018), (iii) assembly of mycolic acid layer (Katsube et al., 2007), (iv) acquisition of iron (Pandey et al., 2014a), and (v) biofilm formation (Yaseen et al., 2018). Given its multiple roles, it is not surprising that it has emerged as a key player in influencing Mtb growth *in vitro* and in alveolar macrophages (Sassetti et al., 2003; Pandey et al., 2014a). Consequently, it has been explored as a novel therapeutic target (Bhowmick et al., 2014; Peraman et al., 2021).

HupB, a ~22 kDa protein, harbors N- and C-terminal domains. While the N-terminal (1-108 amino acids, AAs) DNA-binding domain (Bhowmick et al., 2014; Gupta et al., 2014; Ghosh et al., 2016) holds significant homology to other bacterial histone-like proteins (Kumar et al., 2010; Pandey et al., 2014a), the C-terminal (108-214 AAs) tetrapeptide motif (^159^KATKSPAK^166^) containing-domain is identical to eukaryotic histone H1/H5 proteins (Prabhakar et al., 1998; Gupta et al., 2014). Although the N-terminal domain directly interacts with DNA, the C-terminal domain is proposed to influence DNA sequence-specific binding (Kumar et al., 2010). Thus, employing both domains, HupB binds AT-rich sequences prominently localized to promoter regions (Bhowmick et al., 2014; Pandey et al., 2014b; Datta et al., 2019). Given this ability, HupB is considered to influence global gene expression by acting as a transcriptional regulator (Bhowmick et al., 2014; Pandey et al., 2014b; Kriel et al., 2018; Datta et al., 2019). Despite its proposed role, its influence on global gene expression is subjected to control by post-translational modifications (such as phosphorylation and acetylation) of its N-terminal domain (Gupta et al., 2014; Ghosh et al., 2016; Sakatos et al., 2018).

Interestingly, an ortholog of HupB, *viz*., Hlp (80% AA identity), that is encoded by an avirulent environmental mycobacterium, *M. smegmatis* (Msm), plays a significant role in Msm's adaptation to several environmental stresses. Thus, an *hlp* knockout mutant is more sensitive to UV, cold shock, and exposure to isoniazid (INH) (Shires and Steyn, 2001; Katsube et al., 2007; Mukherjee et al., 2009; Whiteford et al., 2011). Furthermore, *hlp* expression is significantly increased during the abovementioned stresses and under anaerobic-induced dormancy indicating Hlp's direct role in aiding Msm to negotiate and overcome different stresses (Lee et al., 1998; Shires and Steyn, 2001; Anuchin et al., 2010).

Coincidentally, Mtb also exhibits significantly increased expression of *hupB* upon exposure to INH and during non-replicating persistence (Betts et al., 2002; Reddy et al., 2009; Zhu et al., 2013). Given the above observations and HupB's proposed role as a transcriptional regulator (Bhowmick et al., 2014; Pandey et al., 2014b; Kriel et al., 2018; Datta et al., 2019), we hypothesized that HupB also plays an important role in adapting Mtb to host- and antibiotic-mediated stresses, and that its loss or inactivation makes Mtb highly sensitive to such stresses. Our macrophage infection and *in vitro* culture studies show that while the *hupB* knockout mutant (hereafter referred to as KO) is highly sensitive to these stresses, Mtb (H37Rv; hereafter referred to as WT) robustly thrives. Promisingly, the KO mutant succumbs to even low doses of INH and RIF. In contrast, *hupB* over expression enhanced Mtb tolerance to INH. Comparative surface lipid profiling shows reduced accumulation to loss of several important polar and apolar lipids in the KO mutant that leads to its increased permeability to SDS. Importantly, we show that targeting HupB sensitizes WT to enhance killing by INH and reduces MIC to INH. Thus, our study for the first time demonstrates that not only targeting HupB significantly enhances Mtb's susceptibility to host-mediated stresses and INH- and RIF-mediated killing but also that such a killing is rapid and requires reduced amounts of INH and RIF. In summary, we show that Mtb's HupB is a compelling therapeutic target especially for short-term combinatorial treatment with INH and RIF.

## Materials and methods

### Bacterial strains and their growth conditions

The mycobacterial strain, *viz., Mycobacterium tuberculosis* H37Rv (WT) (Supplementary Table 1) and its derivatives were grown *in vitro* as indicated in Sharma et al. (2019). Briefly, they were cultured at 37°C in Middlebrook 7H9 broth (BD, United States) or Middlebrook 7H11 agar (BD) supplemented with (i) OADC (BD; 1 × as final, 10 × OADC – as stock; (ii) 0.2–0.5% of glycerol (for broth, 0.2%; for agar, 0.5%), and (iii) 0.05% Tween 80 (Merck, United States). *Mycobacterium smegmatis* mc^2^155 (Msm) (Supplementary Table 1) was grown *in vitro* at 37°C in the abovementioned media except that 1× ADC replaced 1 × OADC (Garces et al., 2010). *Escherichia coli* (*E. coli*) strains, *viz*., DH5α, HB101 (used for cloning; Supplementary Table 1; Thermo Fisher Scientific, United States), and BL21-DE3 with pLysS (used for *hupB* overexpression and antibody generation) (Supplementary Table 1) were grown in Luria-Bertani (LB) broth/agar (HiMedia, India) according to Sezonov et al. (2007). The final concentrations of antibiotics used (either for maintaining plasmids and/or supporting growth) were as follows: for mycobacteria: hygromycin 50 μg/ml (Thermo Fisher Scientific), cycloheximide 50 μg/ml (Thermo Fisher Scientific), and kanamycin 25 μg/ml (Merck) and for *E. coli*: chloramphenicol 25 μg/ml (Merck), kanamycin, 100 μg/ml (Merck), and hygromycin 150 μg/ml (Merck).

### Cloning

To clone *hupB* into pET28a (pNA1; Supplementray Table 2), using the genomic DNA of Mtb as the template, a primer pair, KAP403F and KAP404R (Supplementary Table 3), and a Phusion Taq polymerase (Thermo Fisher Scientific), we PCR-amplified *hupB* and digested the eluted (HiMedia) amplicon with NdeI (NEB, United States) and BamHI (NEB). The digested and purified amplicon was ligated [using T4 DNA ligase (NEB)] to similarly digested pET28a to obtain 6X-His*hupB*. The episomal expression vector pVV16 (Yaseen et al., 2018) containing *hupB* (pNA4; Supplementary Table 2) was from our lab collection. To clone *hlp*, using the genomic DNA of Msm as the template, a primer pair, KAP634F and KAP635R, (Supplementary Table 3), and a Phusion Taq polymerase (Thermo Fisher Scientific), we PCR-amplified *hlp* and digested the eluted (HiMedia) amplicon with EcoRI (NEB) and HindIII (NEB). The digested and purified amplicon was ligated [using T4 DNA ligase (NEB)] to similarly digested pVV16 to obtain pVV16+*hlp* (pNA5; Supplementary Table 2). The molecular construction of pNA2 and 3 is provided in the subsection “KO generation and its complementation” (refer below).

### Antibody generation and western analyses

BL21 DE3 (pLysS) *E. coli* strain harboring 6X-His: *hupB* (pNA1; Supplementary Table 2) was induced with 0.5 mM isopropyl β-D-1-thiogalactopyranoside (Merck) at 37°C for 4 h. The induced cultures were pelleted down at 4°C and 14,000 rpm. The washed pellet (1X PBS, pH 7.4) was boiled in 1 × Laemmli buffer for 15 min at 95°C and electrophoresed on 15% SDS-PAGE gels and evaluated for overexpression by coomassie staining and anti-His antibody (Ab). Since, both HisPur cobalt and Ni-NTA beads non-specifically retained several contaminating *E. coli* proteins, we followed the protocol as reported in Atmakuri et al. (2003) for generation of polyclonal Ab specific to HupB. Briefly, we cut the overexpressed band out, eluted the proteins within, and generated polyclonal Ab to the eluted mix in rabbits (outsourced to TheraIndx Lifesciences, India). We verified the specificity of the generated Ab by Western blot analysis (Mahmood and Yang, 2012) of whole cell protein lysates of Msm and Mtb using pre-bleed sera of the rabbits as negative control. We further purified the obtained antisera using the method described in Atmakuri et al. (2003). We immediately neutralized the purified Ab eluate with few drops of 1 M Tris, pH 7.5 (tested for pH on pH paper, HiMedia), and stored it as aliquots with 0.1% bovine serum albumin (BSA; Bio Basic, Canada) and 0.02% sodium azide (Merck) for future use. Total proteins in different samples were quantitated using a BCA (bicinchoninic acid) kit (Thermo Fisher Scientific). HupB-specific protein bands were identified by Western blot analysis using purified polyclonal anti-HupB Ab. Western blots were developed using SuperSignal^TM^ West Femto Maximum Sensitivity Substrate (Thermo Fisher Scientific) and HupB-specific signals monitored using a ChemiDoc^TM^ MP Imaging system (Bio-Rad, United States). Image Lab version 6.0.1 (Biorad) was used for semi-quantitation of the HupB-specific bands. The purified anti-HupB Ab could also detect Hlp-specific bands (panel C-ii of Supplementary Figure 3), as HupB and Hlp are orthologs with a significant amino acid identity (~80%).

For assessing HupB protein levels (by Western blot analyses) across different phases of growth, Mtb grown in either rich (7H9 media containing 10% OADC and 0.05% Tween-80; Garces et al., 2010) or minimal media Sauton's broth, (Parish and Stoker, 2001) were pelleted down at 4,000 rpm and 4°C for 15 min and washed with cold 1 × PBS, pH 7.4. The pellets were then resuspended in a 400 μl bead beating buffer (0.1 M Trizma (pH 6.8) with 1 mM EDTA; Merck) with 1 × protease inhibitor cocktail (Thermo Fisher Scientific). The suspension was lysed with bead beating (Biospec Products, United States) for 8 cycles (each 45 s) with 2-min incubation between the cycles on ice. The obtained lysed suspension was spun down at 14,000 rpm for 10 min at 4°C. In 75 μl of total lysate, 25 μl of 4× Laemmli's buffer (Green and Sambrook, 2012) was added, and the mix was boiled at 95°C for 15 min. The boiled lysate was spun down for 5 min at room temperature (RT) at 14,000 rpm, and total proteins were estimated. Equal amount of proteins were resolved on 15% SDS-PAGE for western analyses with anti-GroEL2 (BEI Resources, United States) and anti-HupB (this study) antibodies. The same gel was cut into two halves; the top half was developed for evaluating GroEL2 protein levels, and the bottom half was developed for monitoring HupB protein levels.

### KO generation and its complementation

We employed a temperature-sensitive mycobacteriophage-based gene knockout strategy (Bardarov et al., 2002) to generate KO. To clone the immediate flanking regions to *hupB* (i.e., 798 bp upstream and 800 bp downstream) using the genomic DNA of Mtb as the template, primer pairs (KAP475F and KAP476R upstream, and KAP477F and KAP478R downstream; Supplementary Table 3), and a Phusion Taq polymerase, we PCR-amplified each of the flanking regions and digested the eluted (HiMedia) amplicons with HindIII and NheI (for upstream) and XbaI and BspHI (for downstream, NEB), respectively. The digested and purified “upstream” amplicon was first ligated (using T4 DNA ligase) to similarly digested cosmid pYUB584 to obtain pYUB584: *hupB* upstream (pNA2; Supplementary Table 2). Then, the digested and purified “downstream” amplicon was ligated (using T4 DNA ligase) to similarly digested pNA2 to obtain pNA3 (Supplementary Table 2). Clone pNA3 was verified by restriction digestion as well as by sequencing (both upstream and downstream flanks). pNA3 was then digested with PacI, gel eluted, and ligated overnight (O/N) (with T4 DNA ligase at 16°C) with similarly digested phage λDNA. The ligation mixture was then transformed into the *E. coli* strain HB101 (Supplementary Table 1), and positive clones were selected on hygromycin-containing LB agar plates (pNA6; Supplementary Table 2). The DNA representing pNA6 was extracted using a plasmid DNA extraction kit (MDI). Subsequent steps of packaging, transformation, transduction, and phage propagation were as described in Bardarov et al. (2002). We ensured that the phage titers were ~10^8^-10^9^ PFU/ml. After checking the sensitivity of phages obtained at 37°C, they were transduced into WT mycobacteria (grown to ~1 OD_600_, washed in MP buffer (contains 50 mM Tris Cl (pH 7.4), 150 mM NaCl, 10 mM MgSO_4_, 2 mM CaCl_2_) and resuspended in ~1 ml of rich media. The transduced culture was incubated O/N without shaking at 37°C. Then, the mix was recovered in ~5 ml of the rich media and incubated O/N at 37°C with continuous shaking (150 rpm). The culture was finally plated on 7H11 (supplemented with 50 μg/ml hygromycin) plates with 10% OADC and 0.05% Tween-80 and incubated at 37°C. Colonies that appeared after 4–6 weeks were screened for KO by PCR.

Putative KO candidates were confirmed by Western blot analysis using a purified anti-HupB antibody. The KO was further confirmed by Sanger sequencing of junction flanks, remaining portions of *hupB* ends, and the inserted hygromycin-resistant gene cassette. The KO was then complemented with either *hupB* (pNA4; Supplementary Table 2) or *hlp* (pNA5; Supplementary Table 2), and the complementation was verified by Western blot analysis with the purified anti-HupB antibody.

### Mtb and *E. coli* transformation

WT and KO Mtb strains were transformed as per standard protocol (Parish and Stoker, 2001). Briefly, using Gene Pulser Xcell (BioRad) and required plasmid DNA (~350 ng), freshly made electrocompetent cells were transformed as per Sharma et al. (2019). All *E. coli* transformations were as per standard protocol (Sambrook and Russell, 2006) that involved use of CaCl_2_ competent cells with 60 s heat shock at 42°C.

### RNA isolation and real-time PCR

The RNA from Mtb strains was isolated as described in Sharma et al. (2019). Approximately 2 × 10^9^ WT and KO mycobacterial cells were used for RNA extraction. The RNA was isolated from the WT and KO pellets with a DNA, RNA, and protein purification kit (Machery-Nagel NucleoSpin^TM^; Germany) as per the manufacturer's protocol. Three μg of the eluted RNA were treated with 1 μl of Turbo DNase enzyme (Turbo DNA-free Kit; Thermo Fischer Scientific, United States) to avoid contaminating the genomic DNA. Two μg of the DNase-treated RNA was used as template to generate cDNA as per PrimeScript 1st strand cDNA synthesis kit (Takara, Japan). One μl of the generated cDNA for each sample was taken for quantitative real-time PCR (qRT-PCR) using 5 × HOT FIREPol Evagreen qPCR Mix Plus (SYBR Green; Solis Biodyne, Estonia) on the Stratagene mx3005p system (Agilent Technologies, United States). The primer pairs used are indicated in Supplementary Table 3. *sigA* transcript levels (Ct value) in different strains were used as internal controls for normalization and accurate estimation of Ct values of all genes under study.

### Simulating host-induced and antibiotic stresses for western blot analysis

Freshly grown mycobacterial cultures (~ 1 OD_600_ in 10 ml rich media) were washed once with fresh sterile 10 ml rich media and sub-cultured to 0.05 OD_600_ (by taking the required aliquot) in 50 ml fresh rich media. When OD_600_ reached ~0.2 −0.3, cells were washed twice in fresh rich media (control) or in media used for inducing stress. Then, pellets were resuspended in 50 ml of appropriate media used for stress. Sublethal concentrations of antibiotics and molecules were employed to induce stress. For oxidative stress, 5 mM H_2_O_2_ (Merck, United States) was added, and stress was imposed for 72 h. For nitrosative stress, 1 mM sodium nitrite (Merck, United States) (pH 5.2, rich media) was added, and stress was imposed for 72 h. For pH stresses, the rich medium was adjusted to required pH (6.4, 5.6 and 4.2, with concentrated HCl; Merck), and stress was imposed for 72 h. For nutritional depletion, bacteria were conditioned for two generations in Sauton's medium (Garces et al., 2010) and then grown from 0.05 OD_600_ to different phases of growth.

Antibiotic-mediated stress imposition was achieved with 2.91 μM of INH (Merck), 6 nM of RIF (Merck), and 4.9 μM of ethambutol (EMB; Merck) in 50 ml of 0.4 OD_600_ culture (subcultured from ~0.4 to 0.6 OD_600_ culture). Five days post treatment, cells were washed twice with cold 1× PBS (pH 7.4), and bacteria were pelleted down for 15 min at 4,000 rpm and 4°C. Proteins (for Western blot analyses) and RNA (for real-time PCRs) were extracted from the pellets (described earlier).

### AlamarBlue assay for MIC determination

We followed the protocol of Collins and Franzblau (1997) with slight modifications. Briefly, freshly grown mycobacterial cultures (~ 1 OD_600_ in 10 ml rich medium) were washed once with fresh sterile 10 ml rich medium and subcultured to 0.05 OD_600_ in 10 ml fresh rich medium. When OD_600_ reached 0.4–0.6, the cells were washed once with the fresh rich medium at 4,000 rpm and RT for 15 min and then used for estimation of MIC. Approximately 3.75 to 4 × 10^4^ colony-forming units (CFUs) were used for the assays. Two-fold dilutions of different drug concentrations (INH:0.02 to 23.3 μM; RIF:0.75–768 nM; EMB:0.15–156.63 μM) were added in triplicates to different wells with appropriate controls (positive-culture only; negative-medium only, and drug only). The final volume in each well was made up to 200 μl with the rich medium and gently mixed 3–4 times with a 200-μl pipette. After 5 days of incubation at 37°C (without shaking), 22 μl of 10× alamarBlue (HiMedia) was added, and the contents were mixed again before incubation (without shaking) for additional 2 days. Color change from blue (metabolically inactive/dead) to pink (metabolically active/growing) was photographed and recorded. To prevent loss of medium due to evaporation in experimental wells, all the peripheral wells were filled with 200 μl of sterile rich medium.

### Colony morphology and growth curves studies

WT, KO, KO+*hupB*, and KO+*hlp* (Supplementary Table 1) were initially grown in the rich medium (10 ml at 37°C and 150 rpm) to ~1 OD_600_, and then pelleted down (at 4,000 rpm and RT for 10 min), and the pellets washed in 10 ml of the fresh sterile rich medium, For colony morphology, primary cultures were subcultured to 0.05 OD_600_, allowed to grow to 0.2–0.3 OD_600_, and then equal numbers of cells (~ 1.6 × 10^5^CFUs/ml) were spotted on Middlebrook 7H11 agar supplemented with 1× OADC, 0.5% glycerol, and 50 μg/ml cycloheximide. After 45 days of incubation at 37°C, the colony morphology of each was recorded. To monitor *in vitro* growth (axenic cultures in broth) in the rich medium, primary cultures in biological triplicates (as grown above) were subcultured to 0.05 OD_600_, and growth was monitored across different growth phases (lag to stationary).

### Assessment of susceptibility of KO to host-induced and antibiotic stresses

Freshly grown mycobacterial cultures (~0.8 OD_600_ in 10 ml rich medium) were washed once with 10 ml fresh sterile rich medium and subcultured to 0.05 OD_600_ in 50 ml fresh rich medium. When OD_600_ reached ~0.2–0.3, cells were washed twice in the fresh rich medium (control) or in the medium used for inducing stress. The cell pellets were resuspended in appropriate stress medium. A 5-ml culture at 0.25 OD_600_ was used for stress induction. For oxidative stress, 10 mM H_2_O_2_ was added, and stress was imposed for 72 h. For nitrosative stress, 5 mM sodium nitrite (pH 5.2, rich medium) was added, and stress was imposed for 72 h. For pH stress, the rich medium was adjusted to required pH (6.4, 5.6, and 4.2; with concentrated HCl), and stress was imposed for 7 and 14 days. Growth response to each stress was recorded in CFUs.

The antibiotic-mediated stress was as per Singh et al. (2013). Briefly, different mycobacterial strains (0.1 OD_600_) at 5 ml were exposed to different concentrations of INH, RIF, and EMB, and growth was recorded in CFUs on days 7 and 14. For targeting HupB with stilbene (SD1), we employed 100 μM SD1 (as obtained from our MIC study, Supplementary Table 4), in 5 ml culture and recorded growth of bacteria in CFUs on 2^nd^, 6th and 12th days.

### Membrane permeability to sodium dodecyl sulfate

Membrane permeability was assessed as described in Garces et al. (2010). Primary cultures (as grown above) of different mycobacterial strains, *viz*., WT, KO, and KO+*hupB*, were subcultured to 0.05 OD_600_ in 50 ml rich medium. When OD_600_ reached 0.2–0.3, cells were washed twice in the fresh rich medium (control) or in the medium used for inducing stress. Membrane permeability was assessed by exposing 5 ml of 0.25 OD_600_ cultures (in biological triplicates) to 0.05% SDS (Merck) for 72 h, and CFUs were recorded by plating cultures 24, 48, and 72 h post exposure.

### Lipid extraction and analyses

Mycobacterial lipids were extracted as previously described by Chauhan et al. (2013), with slight modifications. Briefly, 100 ml of freshly grown (~ 1 OD_600_, as described above) mycobacterial cultures, *viz*., WT, KO, and KO+*hupB*, were pelleted down at 4,000 rpm and RT for 15 min and washed twice in fresh sterile 1 × PBS (pH 7.4), and equal weight of pellets (~14 mg) was heat-killed by exposure to 95°C for 1 h. Apolar lipids were extracted by resuspending the heat-killed pellets in 2 ml of 0.3% sodium chloride (w/v) solution prepared in methanol. To this, 1 ml of petroleum ether was added. The total suspension was thoroughly mixed by gentle shaking O/N on a rotor at RT. The suspension was then spun down for 10 min at RT and 4,000 rpm. The upper layer consisting of apolar lipids was collected in a separate vial. One ml of petroleum ether was added to the lower layer, vortexed, and mixed end-over-end for 15 min. The cell suspension was re-centrifuged to recollect the upper layer. The upper layers comprising apolar lipids were pooled and dried by incubation for 24 h at 37°C.

To the bottom layer, we added 2.3 ml of chloroform: methanol: 0.3% sodium chloride (at 90: 100: 30, v/v/v). The cell suspension was gently rocked O/N (at RT) and then spun down at 2,500 rpm for 10 min. Polar lipids, present in the supernatant fraction, were collected, and the pellet was further treated twice with 750 μl of chloroform: methanol:0.3% sodium chloride (50: 100: 40, v/v/v) to obtain all polar lipids. The supernatants from the three extractions were pooled and further extracted with 1.3 ml of chloroform and 1.3 ml of 0.3% sodium chloride. The lower layer comprising polar lipids was collected into a fresh glass tube and incubated at room temperature until it became dry.

Required amounts of polar and apolar lipids were suspended in chloroform: methanol (2:1, v/v), spotted on TLC plates (Silica gel on TLC Al foil; Fluka, United States), and resolved for visualization of different lipids.

Apolar lipids: equal quantity (for WT and KO+*hupB*) or thrice the quantity (for KO) of phthiocerol dimycocerosate (PDIM) and triacylglycerol (TAG) fractions was separated by 1-dimensional TLC (in petroleum ether:diethyl ether (85: 15), and plates were charred (80°C for 5–10 min on hot plate) and detected by 10% phosphomolybdic acid spray. Similarly, equal quantity (for WT and KO+*hupB*) or thrice the quantity (for KO) of mycolic acid (FAMEs, fatty acid methyl esters, and MAMEs, mycolic acid methyl esters) fraction was separated by 1-dimensional TLC [using solvent hexane: ethyl acetate (95:5)], and plates were charred and detected by 10% phosphomolybdic acid (in ethanol) spray (Chauhan et al., 2013; Sambandan et al., 2013).

Polar lipids: an equal quantity (for WT and KO+*hupB*) or three times the quantity (for KO) of polar lipids was separated by 2-dimensional TLC (size: 10 × 20 cm). First, they were resolved using chloroform:methanol:water (60:25:4) and then later chloroform:acetic acid:methanol:water (40:25:3:6). The plates were then charred (80°C for 5–10 min on hot plate) and detected by 10% phosphomolybdic acid spray (Chauhan et al., 2013).

### Killing Mtb *in vitro* with SD1 in combination with INH

WT cultures (in biological triplicates) were inoculated in 5 ml rich medium at 0.1 OD_600_. Twenty-four hours into growth, SD1 (Chembridge, United States) was added at 100 μM. Twenty-four hours later, (i.e., 48 h from the start of the experiment), different concentrations of INH were added to the cultures. The effect of the combinatorial exposure was measured in CFUs by plating aliquots on days 0, 2, 6, and 12. Appropriate controls included untreated and treated alone with either INH or SD1.

### *In vitro* intracellular macrophage infection and killing assay

Infection assays with macrophages (THP-1) were broadly conducted according to Sharma et al. (2019). Briefly, required volumes of WT, KO, and KO+*hupB* cultures were washed in a filter-sterilized RPMI medium, clumps were removed by filtering through 5-μm sterile membrane filters (MDI), OD_600_ was monitored and then used for infecting ~2 × 10^5^ PMA (Merck)-differentiated THP-1 macrophages at 1:10 MOI. After 4 h of infection, extracellular bacteria were killed with Amikacin (200 μg/ml), and macrophages were lysed at different time points including day 0. The lysates obtained were plated on 7H11 supplemented with 1× (10%) OADC, 0.5% glycerol, and 50 μg/ml cycloheximide.

### MIC_99_ assay

The MIC_99_ of SD1 against WT was determined by broth microdilution (Supplementary Table 4; Arora et al., 2020). For this, two-fold serial dilutions of SD1 were prepared on 96-well clear “U” bottom plates followed by addition of ~50 μl culture of 1:1,000 times diluted early logarithmic WT culture (OD_600_ ~0.2). The plates were incubated without shaking at 37°C for 14 days. The lowest concentration of SD1 at which no round pellet formation was visually (using magnifying glass) observed was considered as the MIC_99_ value. The assay plates included controls, *viz*., medium only and INH alone.

### *In vitro* checkerboard analysis for INH and SD1 drug combination study

An *in vitro* drug combination of INH and SD1 was generated by two drug checkerboard assays on 96-well clear “U” bottom plates (Supplementary Table 5; broth microdilution method; Arora et al., 2020). SD1 was diluted horizontally, and the first line TB drug (INH) was diluted vertically to make various drug combinations. Furthermore, the early logarithmic culture (~0.2 OD_600_) of WT was diluted 1:1,000 times, and ~50 μl was added to the above plates followed by incubation at 37°C for 14 days. Fractional inhibitory concentration (FIC) and fractional inhibitory concentration index (FICI) were calculated for various concentrations of the drug combinations as in Arora et al. (2021).

## Results

Given the various properties of HupB and/or its homolog Hlp (Prabhakar et al., 1998; Katsube et al., 2007; Kumar et al., 2010; Bhowmick et al., 2014; Gupta et al., 2014; Pandey et al., 2014a; Ghosh et al., 2016; Hołówka et al., 2017; Kalra et al., 2018; Yaseen et al., 2018; Datta et al., 2019), its expression *per se* being modulated under low iron conditions (Pandey et al., 2014b), non-replicating persistence (Lee et al., 1998; Shires and Steyn, 2001; Betts et al., 2002; Anuchin et al., 2010), and upon exposure of Mtb to INH (Reddy et al., 2009; Whiteford et al., 2011; Hadizadeh Tasbiti et al., 2016; Sakatos et al., 2018; Arora et al., 2021; Hadizadeh Tasbiti et al., 2021), we hypothesized that to survive and respond to different host- and antibiotic-mediated stresses, Mtb employs HupB as a key player.

### HupB protein levels significantly alter in response to Mtb exposure to different host-mediated and antibiotic-induced stresses imposed *in vitro*

**Figure 1:**
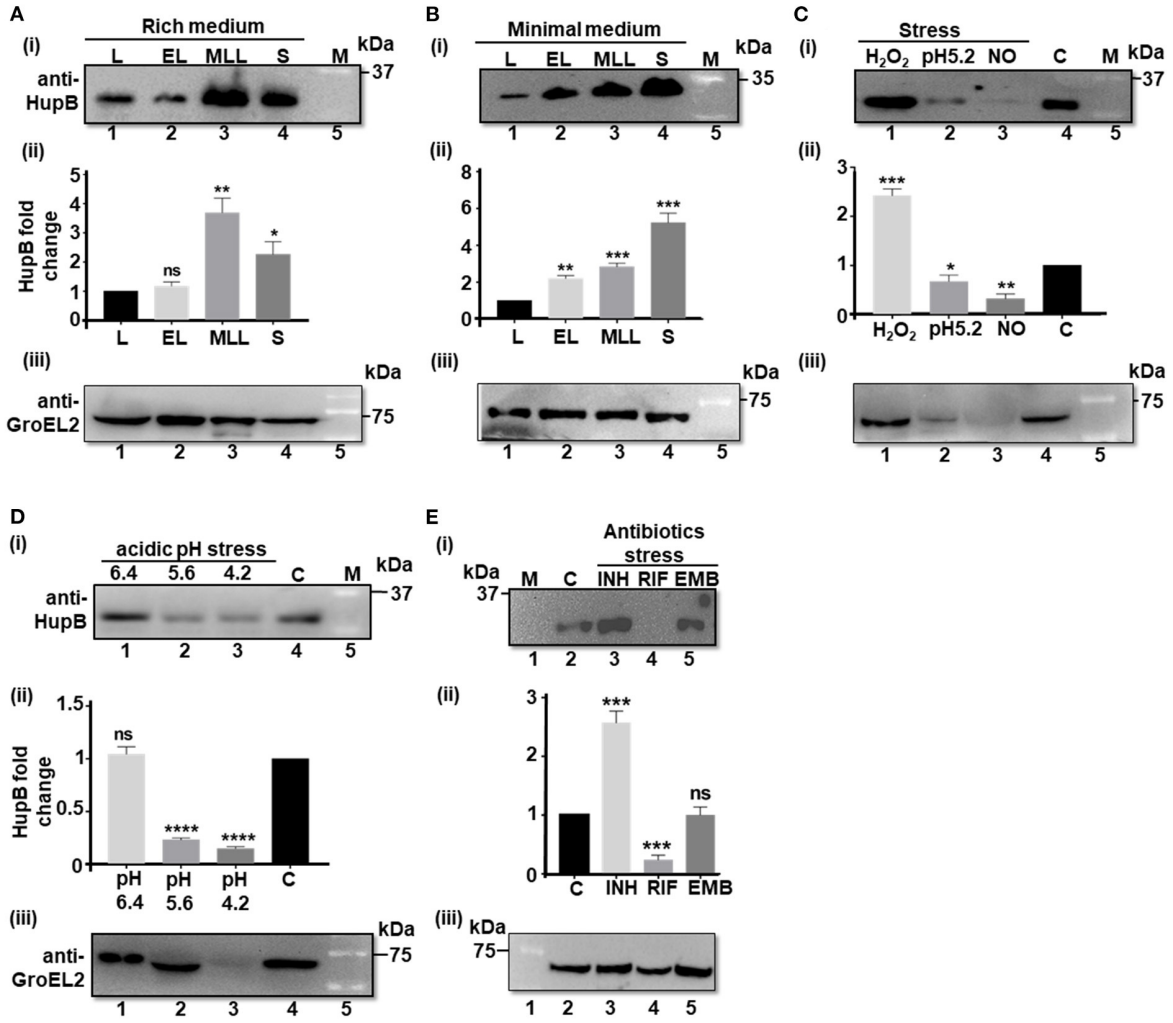
HupB protein levels significantly alter in response to Mtb exposure to different host-mediated stresses imposed *in vitro*. **(A)** WT (H37Rv) grown in different phases in rich (7H9 + 1 × OADC +0.05% Tween-80), and **(B)** nutrient-depleted/minimal media (Sauton's) or **(C–E)** grown under different stress conditions (details are shown in Materials and methods^#^) was pelleted down, washed, and lysed by bead beating. Equal quantity of lysate proteins (~ 30 μg; also refer to Supplementary Figures 1, 2) was boiled for 15 min in 1 × Laemmli sample buffer and resolved (in 15% SDS-PAGE). Western blot analyses were performed with a purified polyclonal anti-HupB antibody [(i) of **(A–E)**] and a polyclonal anti-GroEL2 antibody [(iii) of **(A–E)**]. The intensity of HupB bands was semi-quantified with a densitometer scan (GelDoc, BioRad) and plotted as HupB fold change [Y-axis; (ii) of **(A–E)**]. **(A,B)** L, lag phase; EL, early Log phase; MLL, mid to late log phase; S, stationary phase. **(C)** H_2_O_2_, oxidative stress imposed for 3 days (d) with 5 mM hydrogen peroxide; NO, nitrosative stress imposed for 3 days with 1 mM sodium nitrite at pH 5.2 (rich medium). **(D)** pH stress (pH 6.4, 5.6, and 4.2) imposed for 3 days in the rich medium. **(E)** INH, isoniazid (2.91 μM); RIF, rifampicin (6 nM); EMB, ethambutol (9.79 μM). All antibiotic exposures were for 5 days. kDa, kilo Daltons; C, control (no treatment, bacteria grown in rich medium); M, reference protein marker [for **(B)** (i) - Genedirex protein marker (Cat # PM007-0500) and for (i) of **(A,C–E)**, BioRad protein marker (Cat # 1610373), respectively]; numericals immediately underneath Western blots i.e., (i) and (iii) of **(A–E)** indicate lanes. (ii) of **(A–E)** represents HupB protein level fold change (Y-axis) determined from three independent (biological triplicates) Western blots (only one shown here); control, bacteria grown in rich medium, plotted as fold change value “1.” Significance evaluated by Student *t*-test (comparative analyses with control). *p < 0.05, **p < 0.01, ***p < 0.005, and ****p < 0.001; ns non-significant. All the experiments were performed at least three independent times. The semi-quantitations of the protein bands [as in (ii) of **(A–E)** are thus an average of semi-quantitation performed on images from all the three independent experiments (biological triplicates)]. The best representative blots are shown in this Figure. ^#^For protein analyses, Mtb had to be subjected to sub-lethal doses such that the stress is imposed but bacteria do not die.

Prior to testing our hypothesis, using the WT, we first determined the basal levels of the HupB protein (and as a control we monitored GroEL2 protein levels) in different phases (i.e., lag, early log, mid to late log, and stationary) of Mtb growth *in vitro*, in both rich and nutrient-depleted/minimal (Sauton's, pH 7.4) media (Figures 1A,B; Supplementary Figure 1A). Upon resolving an equal amount of whole cell lysate proteins on denaturing polyacrylamide gels (Supplementary Figure 1B), we observed that, compared to the lag (also used here as reference/control) and early logarithmic (log) phases (Figures 1A,B, respectively), HupB protein levels significantly increased by 2–4 folds in the mid to late log and stationary phases of growth (Figures 1A,B, respectively). Furthermore, in comparison to the rich medium wherein HupB protein levels peaked in the mid to late log phases but dropped by a fold during the stationary phase of growth (Figure 1A), in the nutrient-depleted/minimal medium, HupB protein levels peaked all the way from the lag to the stationary phase of growth (Figure 1B). As expected, under these conditions, GroEL2 levels remained significantly unaltered (Figures 1A,B).

Then, to test our hypothesis, we exposed the WT *in vitro* to different host-mediated *viz*., nutrient depletion (Garces et al., 2010), acidic pH (Singh et al., 2013), oxidative stress and nitrosative stress (Voskuil et al., 2011), and antibiotics-mediated stresses (Singh et al., 2013), and monitored their HupB protein levels by Western blot analyses [Figures 1C–E; (loading controls-panel (i) of Supplementary Figures 2A–C)]. To thrive and establish its niche in alveolar macrophages, Mtb co-opts phagosomes (Pethe et al., 2004; MacGurn and Cox, 2007; Ehrt and Schnappinger, 2009; Huynh et al., 2011). It overcomes oxidative and nitrosative stresses and variation in phagosomal pH (from 6.4 to 4.2) during phagosomal maturation (Pethe et al., 2004; MacGurn and Cox, 2007; Ehrt and Schnappinger, 2009; Huynh et al., 2011; Voskuil et al., 2011; Vergne et al., 2015; Zulauf et al., 2018).

We first ensured that the imparted stress conditions significantly induced the expression of a universal stress protein, Rv2005c [over “no stress” as control (Hingley-Wilson et al., 2010), panel (ii) of Supplementary Figure 2] and altered growth (OD_600_) as expected (panel (iii) of Supplementary Figure 2). Then, we compared the HupB protein levels across different stresses (again by resolving equal amount of total Mtb proteins-panel (i) of Supplementary Figures 2A–C) to “no stress (at pH 7.4/control)” condition used as reference/control (Figures 1C–E). Under different host-mediated stresses, HupB protein levels significantly altered (Figures 1C,D). Under oxidative stress *in vitro*, HupB protein levels significantly increased by approximately two folds (Figure 1C), while under nitrosative conditions, its levels significantly decreased (~5 folds; Figure 1C). HupB levels also significantly lowered (~2–5 folds) at pH 5.6 (Figure 1D) and 4.2 (Figures 1C,D). However, HupB levels remained unaltered when pH was reduced from 7.4 to 6.4 (Figure 1D). Imparting nitrosative stress with sodium nitrite requires reducing medium pH to 5.2. Under these conditions, HupB levels reduced by ~0.5 to 1 fold (Figure 1C). Again, as expected, although GroEL2 protein levels under most stresses remained significantly unaltered, under acidic stress conditions (pH 5.2 and 4.2), its levels were significantly low (Figures 1C,D).

Interestingly, upon exposing the WT to first-line antibiotic INH, we observed that HupB protein levels significantly increased by ~1.5 to 2 folds (Figure 1E). On the contrary, its levels significantly reduced (by 3–4 folds) in the presence of RIF (Figure 1E). However, upon exposure to EMB, despite showing a significant increase in the expression of Rv2005c (Supplementary Figure 2), Mtb did not alter HupB levels (Figure 1E). Again, GroEL2 protein levels remained largely unaltered except for a marginal decrease in the presence of RIF (Figure 1E).

### Loss of *hupB* significantly restricts Mtb growth *in vitro*

Given that HupB protein levels significantly modulated under different host-induced stresses (Figure 1), we examined how Mtb would respond to the same stresses: (i) in complete absence of *hupB* and (ii) when HupB is pharmaceutically inactivated.

**Figure 2:**
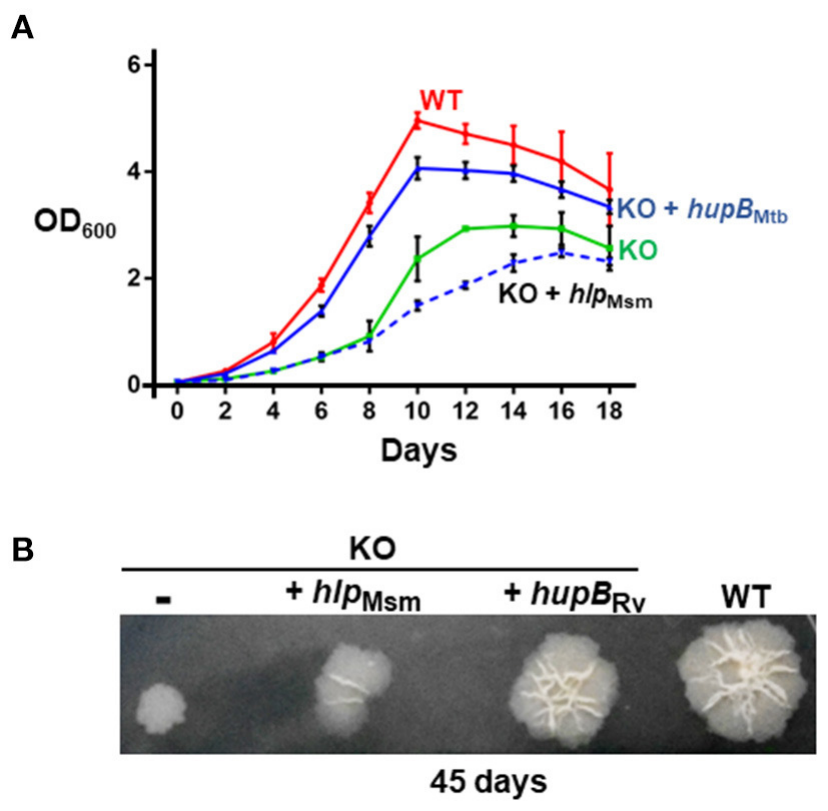
Loss of *hupB* significantly restricts Mtb growth *in vitro*. WT (red), KO (green), KO+*hupB* (blue), and KO+*hlp* (blue-dashed line) were grown *in vitro* in the rich medium, in **(A)** broth (liquid) and on **(B)** agar (solid). **(A)** Growth was initiated at 0.05 OD_600_ in the rich medium and monitored as OD_600_ (Y-axis) for 18 days (X-axis). OD_600_ was recorded in technical duplicates and averaged. Three biological triplicate cultures were monitored for growth. Thus, the OD_600_ plotted (Y-axis) is an average (plus standard deviation) of three OD_600_ values. **(B)** Five μL of 0.05 OD_600_ cultures (approximately equal number of bacterial cells) were spotted on rich medium agar plates, and colony morphology was observed after 45 days. Approximately equal CFUs were spotted (WT 1.65 × 10^5^ CFUs/ml, KO 1.66 × 10^5^ CFUs/ml, KO+*hupB* 1.56 × 10^5^ CFUs/ml, and KO+*hlp* 1.6 × 10^5^ CFUs/ml. The “-” in the 1^st^ spot of **(B)** indicates KO with plain vector.

Employing a mycobacteriophage-based homologous recombination approach, we generated a *hupB* KO mutant (Supplementary Figure 3). We replaced *hupB* with a hygromycin-resistant gene marker (Supplementary Figure 3A) and confirmed the KO by PCR (panel (i) of Supplementary Figure 3B), Western blot analysis (panels (ii) and (iii) of Supplementary Figure 3B), and sequencing (panels (iv) to (vii) of Supplementary Figure 3B). We also successfully complemented the KO with not only *hupB* but also with *hlp* (Supplementary Figure 3C). Then, by inoculating equal numbers (CFUs/ml) of KO, KO+*hupB*, and KO+*hlp*, we compared their growth *in vitro* over a defined period in both liquid (broth) and solid (agar) rich media. Loss of *hupB* severely restricts KO mutant's growth in both the liquid (Figure 2A) and solid (Figure 2B) rich media. Especially in the solid rich medium, even after 45 d, the KO grew extremely slow when compared to the WT (diameter of colony: ~0.3–0.4 cm for KO vs. 1.5–1.6 cm for WT; Figure 2B). Interestingly, only complementation with *hupB* but not *hlp* restored the KO from its growth-restricted phenotype (both in solid and liquid rich media) to the comparative growth phenotype observed with the WT (Figure 2). Consequently, for all our subsequent experiments, we only used KO+*hupB* as the KO-complemented mycobacterial strain.

### Loss of *hupB* makes Mtb highly susceptible *in vitro* to host-simulated stress conditions

Since loss of *hupB* severely restricted the growth of the KO even in the rich solid medium (Figure 2), we hypothesized that the KO will be much more sensitive to growth when exposed to the host- and antibiotic-mediated stresses that trigger the modulation of HupB protein levels in the WT (Figure 1). To evaluate the influence the host-mediated stresses may have on KO growth, we subjected equal numbers (CFUs/ml) of KO and KO+*hupB* mycobacteria to different host-simulated stresses, again for a defined length of time, and compared their growth (in CFUs) to that observed with similar number of WT bacteria.

As expected and reported earlier (Figure 2), even in the rich medium (at pH 7.4), when monitored at the 7 and 14 d time points, compared to the WT, the KO grew significantly slow (Supplementary Figure 4A). In contrast, under identical conditions, at both time points (7 and 14 d), the KO+*hupB* strain exhibited comparable growth to the WT, indicating successful complementation (Supplementary Figure 4A).

**Figure 3:**
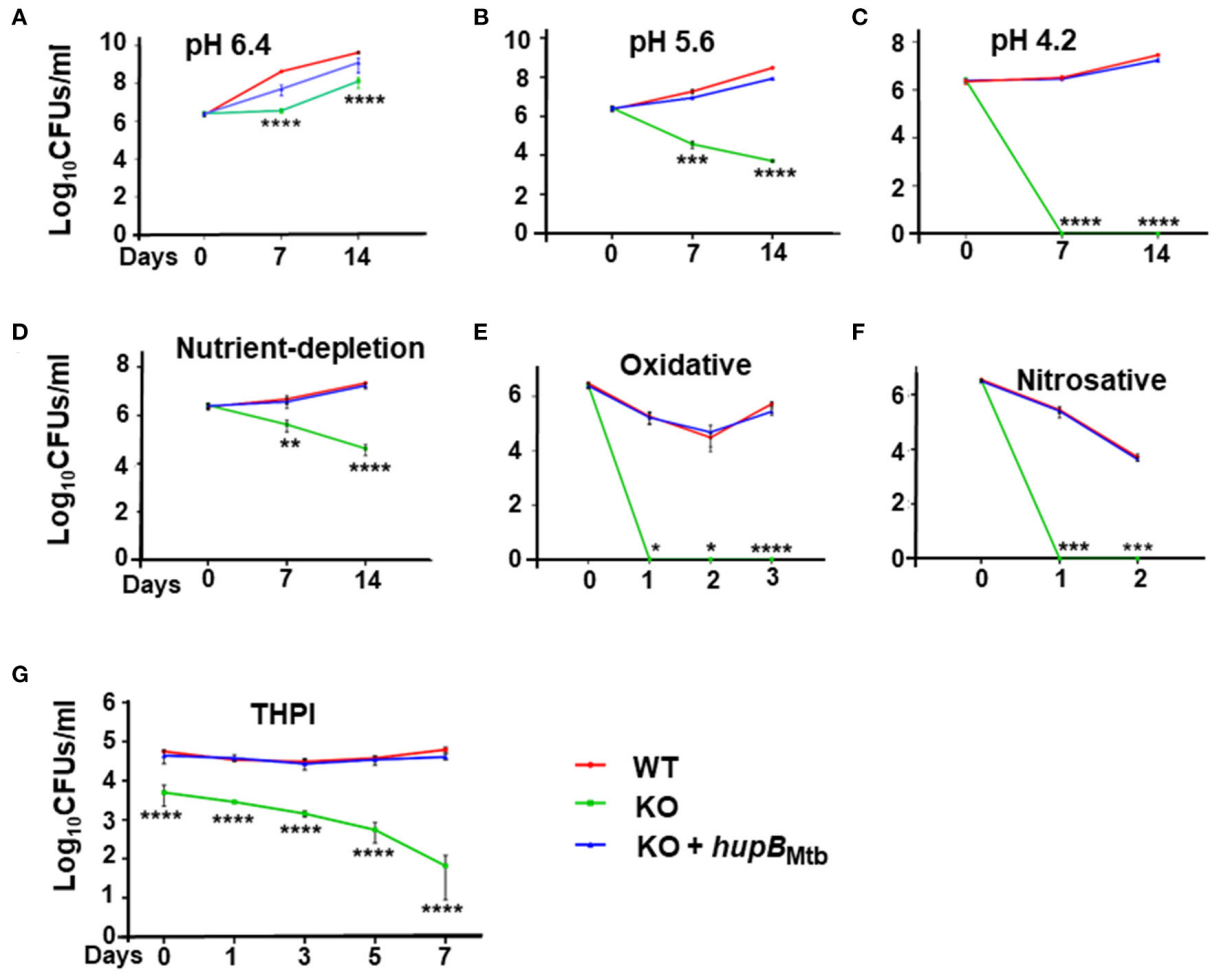
Loss of *hupB* makes Mtb highly sensitive to host-simulated stress conditions. WT (red), KO (green), and KO+*hupB* (blue) were grown in the rich medium to ~0.8 OD_600_, washed, and subcultured at 0.05 OD_600_. When absorbance reached 0.2–0.3 OD_600_, they were washed and normalized to 0.25 OD_600_. Five-ml cultures of each strain were subjected to various forms of host-simulated stress. **(A–C)** pH of the rich medium (pH 7.4) was reduced to pH as denoted; **(D)** medium was changed to 1X TBST (2.5 mM Tris, 4.2 mM NaCl, and 0.05% Tween 80); **(E)** oxidative stress was induced with 10 mM H_2_O_2_; **(F)** nitrosative stress was induced with 5 mM nitric oxide; **(G)** equal number (CFUs/ml) of the WT, KO, and KO+*hupB* cultures were washed in RPMI and used for infecting PMA (phorbol 12-myristate-13-acetate)-differentiated THP-1 macrophages at 1:10 MOI. After 4 h of infection, extracellular bacteria were killed with amikacin (200 μg/ml), THP-1 macrophages lysed with 0.1% Triton-X-100 at different time points (days on X-axis). The lysates obtained were plated on 7H11 + OADC + cycloheximide (50 μg/ml), CFUs were enumerated, and standard deviation values were calculated and then plotted as Log_10_ CFUs/ml (Y-axis). Student *t*-test was performed for comparative analyses, and significance was evaluated. *p < 0.05, ** *p* < 0.01, *** *p* < 0.005, and **** *p* < 0.001. The data are a representation of biological triplicates and technical duplicates.

Upon imposing mild acidic stress, i.e., by reducing pH from 7.4 to 6.4, the growth pattern of the KO remained similar (Figure 3A) to its own exhibited at pH 7.4 (Supplementary Figure 4A). As expected, so did the growth of KO+*hupB* (Figure 3A and Supplementary Figure 4A). However, when we further reduced the pH to 5.6 at both the 7- and 14-day time points, compared to the WT, the KO became more sensitive, and its numbers significantly reduced (by ~2 log; Figure 3B). Interestingly, upon further acidification of the medium to pH 4.2, the KO became extremely sensitive, and it failed to survive even for 7 d (~6 log reduction in numbers; Figure 3C). As expected, the KO also struggled to grow in the nutrient-depleted medium (Figure 3D). By day 14 its numbers (CFUs/ml) significantly reduced (~2 log), indicating again its sensitivity to growing *in vitro* in a nutrient-depleted condition (Figure 3D).

When we subjected the WT, KO, and KO+*hupB* separately to oxidative (Figure 3E) and nitrosative (Figure 3F) stresses, compared to the WT, the KO turned out to be extremely sensitive and failed to survive even for a day (Figures 3E,F). As expected, under all these stresses, similar to the WT, the KO+*hupB* strain continued to proliferate (Figures 3A–F), again indicating successful complementation of KO with *hupB* and the importance of HupB for growth during these stresses. As expected, the aliquots of cells (of these three strains), when used as “no stress controls” for both oxidative (Figure 3E) and nitrosative stresses (Figure 3F), grew similar (Supplementary Figures 4B,C) to their growth observed at pH 7.4 (Supplementary Figure 4A).

Given our observations on KO sensitivity to host-mediated stresses (Figures 3A–F) and since these stresses are imposed by macrophages upon Mtb during infection and survival (Vergne et al., 2015; Zulauf et al., 2018), we also compared the ability of WT, KO, and KO+*hupB* to grow *in vitro* in THP-1 macrophages. Despite using an equal number (CFUs/ml) of bacterial cells for infection, KO numbers reduced immediately post-infection (Figure 3G). As the days progressed, the KO numbers continued to significantly decrease. By day 7, almost 3–3.5 log equivalents of KO bacteria had succumbed to macrophage assault (Figure 3G). In contrast, as expected, even by day 7, similar to WT, KO+*hupB* bacterial cells continued to survive post infection in THP-1. Interestingly, despite using an equal number of KO bacteria for infection, we consistently observed a reduced number (~0.5–1 log) of them infecting THP-1 (Figure 3G).

### Loss of *hupB* makes Mtb highly susceptible *in vitro* to reduced amount of INH and RIF but not EMB

**Figure 4:**
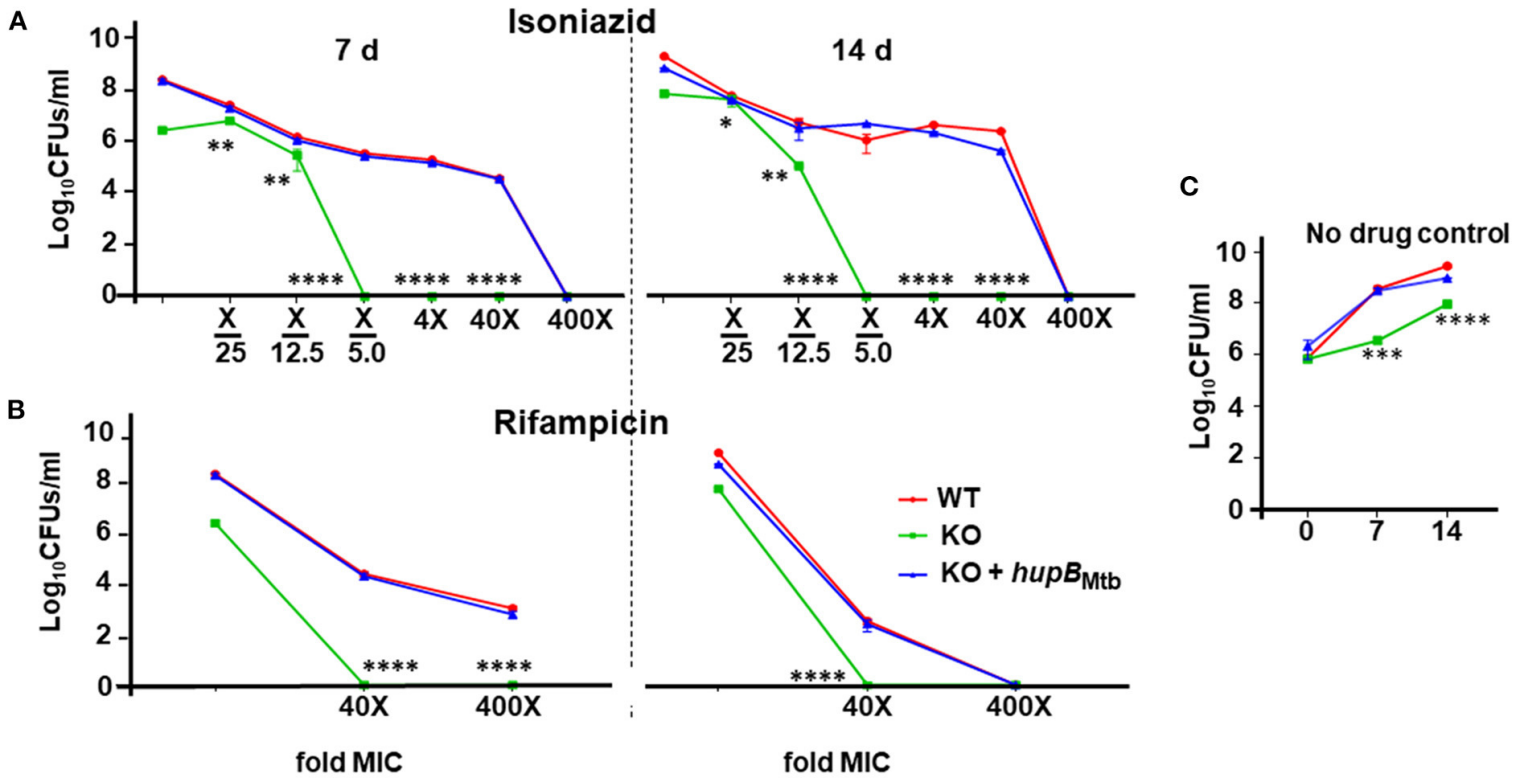
Loss of *hupB* makes Mtb highly susceptible *in vitro* to reduced amount of INH and RIF. WT (red), KO (green), and KO+*hupB* (blue) were separately grown in the rich medium to ~0.8 OD_600_, washed, and subcultured at 0.05 OD_600_. When absorbance reached 0.4–0.6 OD_600_, they were washed and then normalized to 0.1 OD_600_. Five-ml cultures were imparted stress with **(A)** isoniazid and **(B)** rifampicin for 7 and 14 days (separated by a broken black straight line) with varying concentrations (X-axis, denoted as “fold MIC” (minimum inhibitory concentration; MIC denoted as X) and by dilution of surviving bacteria plated on 7H11 + OADC + cycloheximide (50 μg/ml) and enumerated as Log_10_CFUs/ml (Y-axis). MIC for isoniazid and rifampicin is 2.91 μM and 6 nM, respectively. Three biological triplicates were set up, CFUs were enumerated by technical duplicates, numbers were averaged, and SD values calculated were curve-plotted. Student *t*-test was performed for comparative analyses, and significance was evaluated. *p < 0.05, ** *p* < 0.01, *** *p* < 0.005, and **** *p* < 0.001. The data are a representation of biological triplicates and technical duplicates. **(C)** Growth on days 7 and 14 of the three mycobacterial strains [used in **(A)** and **(B)**] without antibiotics (no drug control).

Given that Mtb also experiences stress when exposed to antibiotics (panel (ii) of Supplementary Figure 2C) (Singh et al., 2013; Tiwari et al., 2015), we evaluated the sensitivity of the KO and KO+*hupB* to first-line anti-TB drugs, *viz*., INH, RIF and EMB. Toward that, employing an equal number of KO and KO+*hupB*, we subjected them at varied lengths of time to different concentrations (around their minimum inhibitory concentrations, MICs) of INH, RIF, and EMB, and compared their growth *in vitro* (in CFUs) to that observed with a similar number of WT bacteria (Figure 4). We evaluated the sensitivity of the WT, KO and KO+*hupB* to INH at 1/5^th^, 1/12.5^th^, and 1/25^th^ fold MIC and at 4, 40, and 400 times its MIC [Figure 4A; represented as “X”; MIC for INH is 2.91 μM; MIC determined with alamarBlue Assay on the WT (Supplementary Figure 5); selection of days of treatment and fold MIC were based on preliminary data obtained (Supplementary Figure 6)]. Interestingly, although none of the three strains survived beyond 7 days, when compared to the WT and KO+*hupB*, the KO turned extremely sensitive to INH (Figure 4A). The KO is sensitive to even 1/5^th^ of the MIC (i.e., 0.58 μM), while the WT and KO+*hupB* became completely sensitive only at 400 times of the MIC (i.e., ~1.16 mM).

We then evaluated the sensitivity of the WT, KO, and KO+*hupB* to RIF at 40 and 400 times the MIC [MIC for RIF is 6 nM; MIC determined with alamarBlue Assay on WT; (Supplementary Figure 7A); selection of days of treatment and fold MIC were based on preliminary data obtained (Supplementary Figure 7B)]. Interestingly, while the KO did not survive beyond 7 days at 40 times the MIC (i.e., 0.24 μM), the WT and KO+*hupB* not only required 400 times the MIC (i.e., 2.4 μM) but they also needed an extended period (~14 days) of drug exposure to be killed (Figure 4B).

We finally evaluated the sensitivity of the WT, KO and KO+*hupB* to EMB at 10 and 100 times the MIC (MIC of EMB is 9.79 μM, Supplementary Figure 8A). Despite repeated attempts on different days of exposure, *viz*., 5 (Supplementary Figures 8B,C), 7, and 14 d (Supplementary Figures 8D,E), the KO failed to exhibit significantly higher sensitivity to EMB than the WT and KO+*hupB* (Supplementary Figure 8).

### Overexpression of *hupB* in Mtb significantly enhances MIC *in vitro* to INH and marginally to RIF

Since (i) Mtb's exposure to INH enhances HupB protein levels (Figure 1) and (ii) loss of *hupB* significantly increases Mtb's susceptibility to reduced amount of INH (Figure 4A), we wondered if the MIC to INH might shift in response to *hupB* overexpression. To test this, as well as to evaluate if MIC might also shift for RIF and EMB, we overexpressed *hupB* in Mtb (Supplementary Figure 9), exposed the strain for 5 days to a range of concentrations of INH, and then monitored with an alamarBlue assay for any shift in MIC (Supplementary Figure 9A). Interestingly, compared to the WT, the MIC to INH was increased by ~3 folds (2.91 to 11.6 μM; (Table 1, Supplementary Figure 9A) when HupB protein levels were increased by ~2–3 folds in the WT that overexpressed *hupB* (Supplementary Figure 9B). Surprisingly, although loss of *hupB* makes Mtb more susceptible to low amounts of RIF (Figure 4B), despite several attempts, compared to the WT, we consistently found only a marginal enhancement of MIC to RIF (~ a fold, from 6 to 12 nM) in the Mtb strain overexpressing *hupB* (Supplementary Figure 10). As expected, we did not observe any shift in the MIC to EMB in the *hupB*-overexpressing Mtb strain (Supplementary Figure 11).

**Table 1:** Overexpression of *hupB* in Mtb enhances minimum inhibitory concentration (MIC) *in vitro* to INH.

**Mycobacterial strain**	**INH (μM)**									
WT	0.02	0.05	0.09	0.18	0.36	0.73	1.46	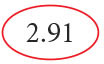	5.82	11.6	23.3
WT overexpressing *hupB*	0.02	0.05	0.09	0.18	0.36	0.73	1.46	2.91	5.82	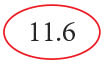	23.3

*WT with vector (plasmid for episomal-based expression) alone (pVV16) (middle row) or with pVV16+hupB (bottom row) was grown in rich medium to ~0.4–0.6 OD_600_, washed, and diluted to 0.0015 OD_600_. Approximately ~3.75 × 10^4^ CFUs of both strains were then exposed to 2-fold increments of INH (μM; middle and bottom rows) for 5 days. Required volume of alamarBlue (to final 1 ×) was added and incubated at 37°C in an incubator for 48 h, color change was recorded, and MIC was determined. Red ovals, MIC (under our conditions). Concentrations below MIC exhibited pink color (Supplementary Figure 9A; indicative of growth and metabolic activity). Concentrations above and including MIC remained blue in color even after 48 h of incubation with alamarBlue (Supplementary Figure 9A; indicative of death and no metabolic activity)*.

### Therapeutic targeting of HupB enhances Mtb's susceptibility *in vitro* to reduced amount of INH

**Figure 5:**
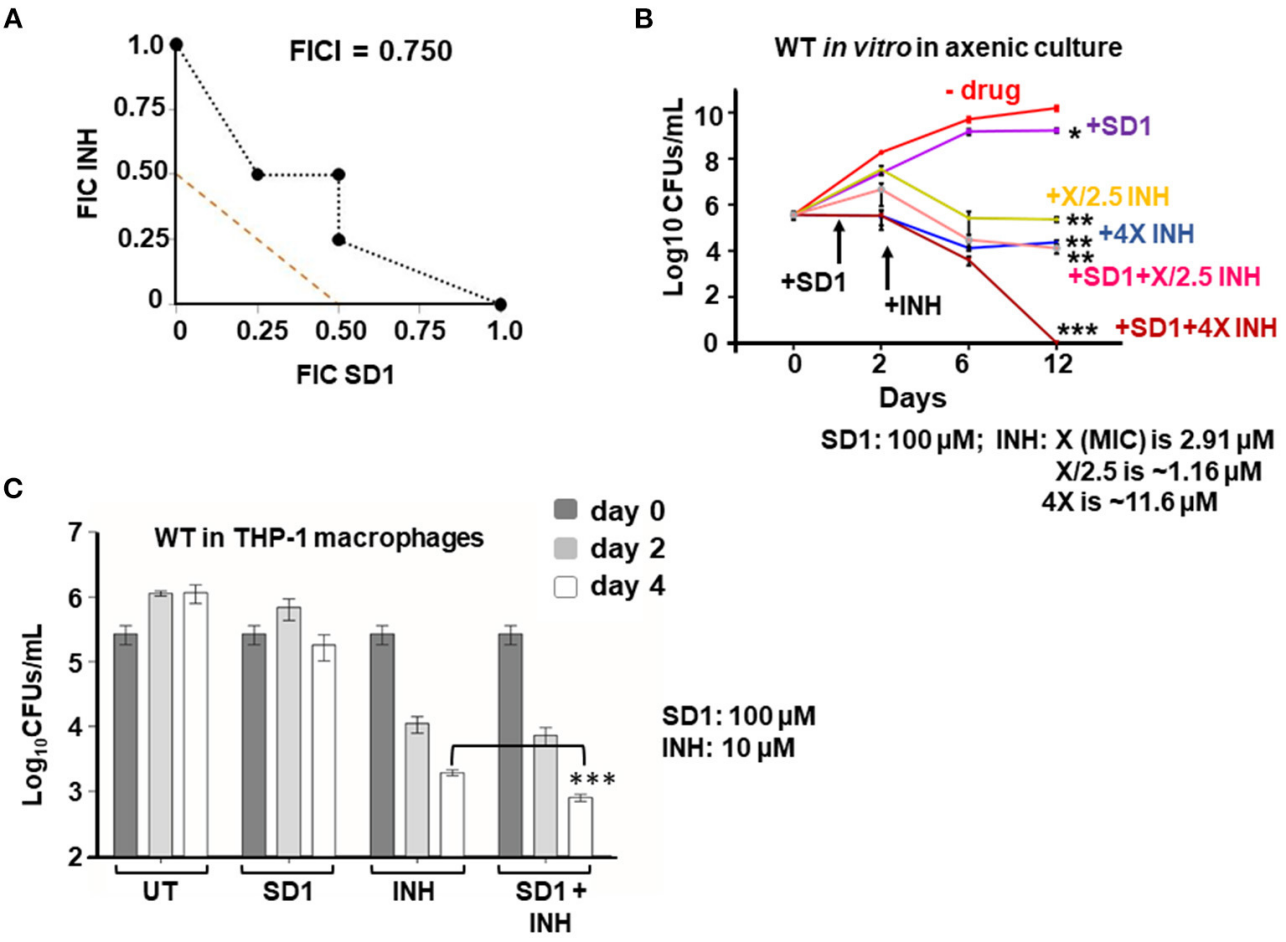
Therapeutic targeting of HupB enhances Mtb's susceptibility *in vitro* to reduced amount of INH. **(A)** Isobolograph reflecting the *in vitro* drug combination of SD1 and INH. Two-drug checkerboard was setup on 96-well “U” bottom plates (broth microdilution method; details in Materials and methods) with two-fold serial dilutions of different concentrations of SD1 (0.39–200 μM) cross-diluted with different concentrations of INH (0.01–0.8 μM). Early logarithmic phase (~0.2 OD_600_) culture of WT was diluted 1,000-fold, and approximately 10^3^ was added to and incubated at 37°C for 10–14 days. FIC (fractional inhibitory concentration) and FICI (fractional inhibitory concentration index) values for their combination were calculated and plotted. **(B)** Evaluation of the combination of SD1 and INH on WT growth *in vitro* and its survival. WT was grown to 0.2–0.3 OD_600_, washed, and cultured to 0.1 OD_600_. Approximately 5-ml culture of the WT was subjected to treatment with indicated concentrations of INH alone or in combination with SD1 (details in Materials and methods). Growth/survival (log_10_CFUs/ml; Y-axis) was monitored for over 12 days (X-axis) and plotted. **(C)** Evaluation of the combination of SD1 and INH on WT growth in THP-1 macrophages. THP-1 macrophages were infected with the WT at an MOI of 1:10, treated with INH alone or in combination with SD1 (at indicated concentrations), and then monitored for bacterial load after 2 and 4 days. Bacteria were collected by lysing THP-1 macrophages with 0.1% Triton-X-100. Their numbers in CFUs were enumerated 3 weeks after plating. The enumerated CFUs were averaged, SD value was calculated and then plotted (log_10_ CFUs/ml; Y-axis). Student *t*-test was performed for comparative analyses, and significance was evaluated. *p < 0.05, ** *p* < 0.01, and *** *p* < 0.005. The data are a representation of biological triplicates and technical duplicates.

Since (i) loss of *hupB* makes Mtb highly susceptible *in vitro* to reduced amount of INH (Figure 4) and (ii) increase in HupB levels of Mtb shifts MIC higher to INH (Table 1, Supplementary Figure 9), we hypothesized that therapeutic targeting of HupB *in vitro* with a reported small molecule inhibitor, *viz*., SD1 (a stilbene derivative), will also increase Mtb's susceptibility to reduced amounts of INH. Employing the broth micro-dilution method (Arora et al., 2020) and using two-fold increments (0.39–200 μM) of SD1, we first determined its MIC on WT to be 100 μM (Supplementary Table 4). To then evaluate if the SD1 and INH combination kills WT faster, employing the micro-dilution method *in vitro*, we evaluated different concentrations of SD1 (0.39–200 μM) and INH (0.01–08 μM, Supplementary Table 5) by checkerboard assay. We observed that as a combination, SD1 and INH marginally shifted the fractional inhibitory concentration index (FICI; Odds, 2003) to 0.75 (Figure 5A) and were able to improve the efficacy of INH by 4-fold (FIC 0.25) as compared to its independent activity (Figure 5A).

**Figure 6:**
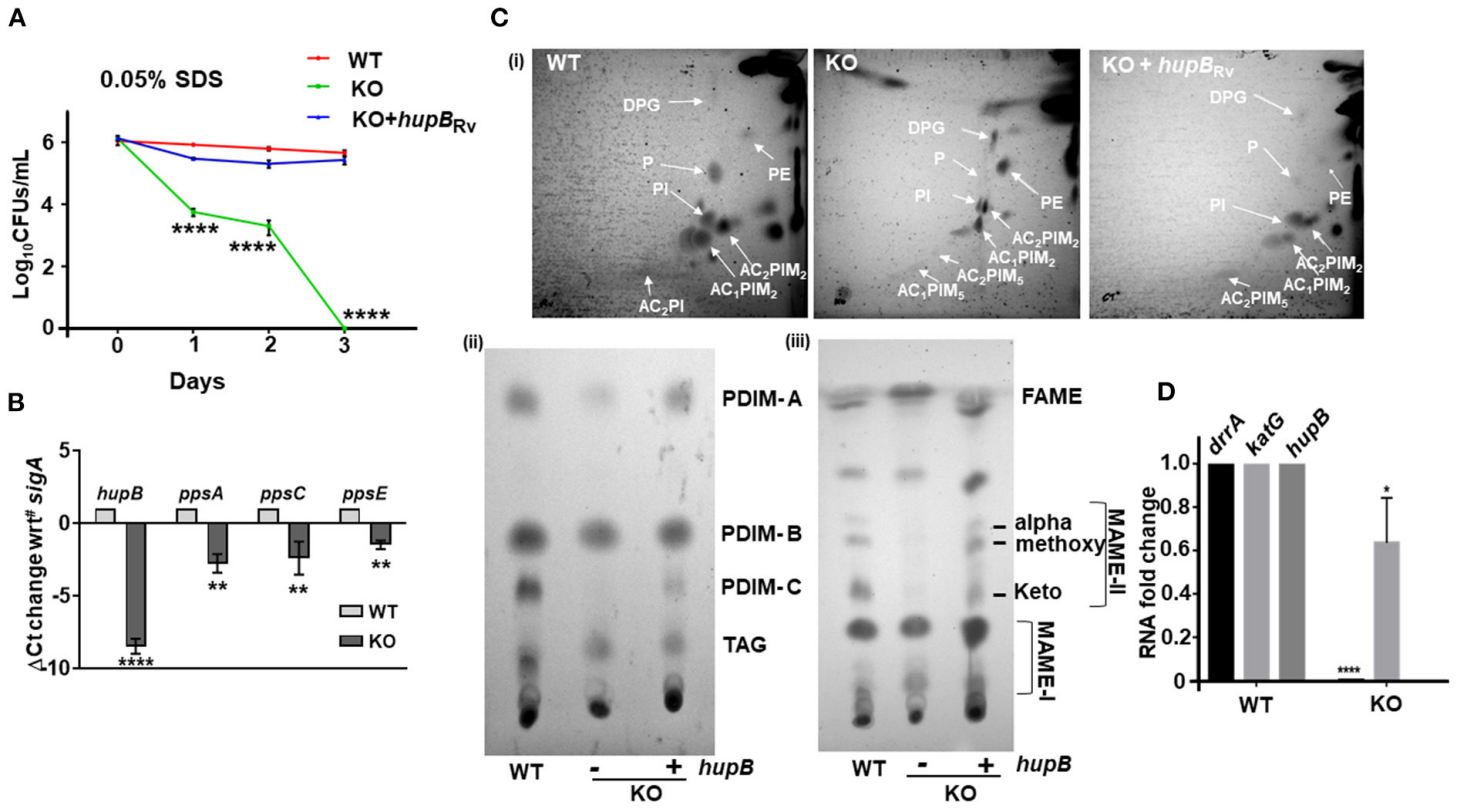
Loss of *hupB* in Mtb alters membrane permeability through reduced expression of polyketide synthases and altered levels of polar and apolar lipids. **(A)** Enhanced permeability of membrane and susceptibility of KO to 0.05% SDS. WT (red), KO (green), and KO+*hupB* (blue) were grown in the rich medium to 0.6–0.8 OD_600_, washed, and subcultured to 0.05 OD_600_. When absorbance reached 0.2–0.3 OD_600_, cells were washed and normalized to 0.25 OD_600_. Approximately 15 ml of cultures of each strain were subjected to stress. Membrane permeability was assessed by exposing bacterial strains (biological triplicates) for 72 h (time on X-axis) to 0.05% SDS. Cultures were plated on appropriate medium, CFUs were enumerated and averaged, and SD values were calculated and then plotted as log_10_ CFUs/ml scale on the Y-axis. Student *t*-test was performed for comparative analyses, and significance was evaluated. **** *p* < 0.001. The data are a representation of biological triplicates and technical duplicates for SDS stress, while in the case of lipid, it is perform in biological triplicates. **(B)** Comparative gene expression analyses of type I polyketide synthases. WT and KO were grown to 0.8 OD_600_ *in vitro*, washed, subcultured to 0.05 OD_600_, and grown to ~1OD_600_, RNA was isolated (from 10-ml cultures), RT-PCR was performed, and ΔCt value of *sigA* was plotted. **(C)** KO exhibits altered levels of polar and apolar lipids. WT, KO, and KO+*hupB* were grown to 1 OD_600_ (100-ml cultures), and total lipids were extracted (details in Materials and methods). [**(C)**-i]: Equal quantity (for WT and KO+*hupB*) or three times the quantity (for KO) of polar lipid fractions from the three strains were separated by 2-dimensional TLC [in chloroform:methanol:water (60:25:4) in the 1^st^ direction and chloroform:acetic acid:methanol:water (40:25:3:6) for the 2^nd^ direction], and then plates were charred (using hot plate) and detected by 10% phosphomolybdic acid spray. White arrows point to specific lipids. DPG, diphosphatidyl glycerol; PI, phosphatidylinositol, PIMs, phosphatidylinositol mannosides, P, phospholipid. [**(C)**-ii]: Equal quantity (for WT and KO+*hupB*) or three times the quantity (for KO) of apolar lipid (PDIM, phthiocerol dimycocerosate; TAG, triacylglycerol) fractions were separated by 1-dimensional TLC [in petroleum ether:diethyl ether (85: 15)], and plates were charred and detected by 10% phosphomolybdic acid spray. [**(C)**-iii] Equal quantity (for WT and KO+*hupB*) or three times the quantity (for KO) of mycolic acid (FAME-fatty acid methyl esters; MAMEs I and II, family of mycolic acid methyl esters) fractions were separated by 1-dimensional TLC [using solvent hexane: ethyl acetate (95:5)], and plates were charred and detected by 10% phosphomolybdic acid (in ethanol) spray (Fontán et al., 2009). **(D)** Comparative gene expression analyses of an efflux pump (*drrA*) and *katG* (catalase peroxidase-peroxynitritase). WT and KO were grown to 0.8 OD_600_ *in vitro*, washed, subcultured to 0.05 OD_600_, and grown to ~1 OD_600_, RNA was isolated (from 10-ml cultures), RT-PCR was performed, and RNA fold change was plotted. *p < 0.05, ** *p* < 0.01. The data are a representation of biological triplicates and technical duplicates.

Despite not being synergistic (synergism requires FICI to be ≤ 0.5; Odds, 2003), because the efficacy of INH was improved by 4-fold (FIC 0.25, Figure 5A), we evaluated their combination on actively shaken axenic cultures of the WT (Figure 5B) and in THP-1 macrophages infected with the WT (Figure 5C). Interestingly, only in the presence of SD1 (100 μM), the WT was more susceptible to even a low amount of INH (Figure 5B). When compared to untreated (plot labeled as “- drug” in Figure 5B), INH, even at 1.16 μM (plot labeled as “+X/2.5 INH” in Figure 5B; X is MIC 2.91 μM), could efficiently reduce WT bacterial numbers from 10^10^ to only 10^6^ (~4-log killing). However, to further kill the WT, just a log more, i.e., from 10^6^ to 10^5^, a 10-fold higher concentration of INH (i.e., 11.6 μM; plot labeled as “+4X INH” in Figure 5B) was necessary. However, surprisingly, such a 5-log killing could be easily achieved with only 1.16 μM INH when bacteria were exposed to INH in combination with SD1 (plot labeled as “+SD1+X/2.5 INH” of Figure 5B). Impressively, an additional 5-log killing (no live bacteria were observed) could be achieved with 11.6 μM of INH only when combined with SD1 (see plot of +SD1+4X INH of Figure 6B).

We also evaluated the SD1 and INH combination on THP-1 macrophages infected with WT bacteria. By day 4, the individual efficacy of INH (~110-fold reduction in intracellular Mtb) was significantly improved to ~240-fold reduction in intracellular Mtb only when INH was used in combination with SD1 (Figure 6C). Importantly, SD1 did not exhibit any cytotoxic effect on THP-1 even at 8 folds its MIC (i.e., at 800 μM, Supplementary Figure 12).

### Loss of *hupB* in Mtb alters membrane permeability through reduced expression of polyketide synthases and altered levels of polar and apolar lipids

Interestingly, Hlp, the ortholog of HupB, also influences cell wall assembly in Msm (Katsube et al., 2007). Since loss of *hupB* makes Mtb (i) struggle with its growth *in vitro* (Figure 2), (ii) turn hypersensitive to several host-induced stresses (Figure 3), and (iii) become highly susceptible to reduced amounts of INH and RIF (Figure 4, Supplementary Figures 6, 7), we speculated that the phenotypes due to loss of *hupB* may be driven by altered surface permeability triggered by loss or reduction of surface lipids. To test this, we first treated an equal number (CFUs/ml) of the WT, KO, and KO+*hupB* with 0.05% SDS, a standard detergent routinely used to test Mtb's membrane integrity (Fontán et al., 2009; Garces et al., 2010) and monitored their growth (in CFUs/ml) over a defined length of time. As expected, WT numbers were barely altered even after 3 days of exposure to SDS (Figure 6A). In contrast, the KO was highly sensitive to the detergent exposure (Figure 6A). Within 1 day, 50% of KO bacteria died. By day 3, none of them survived. As expected, the KO restored its tolerance to SDS only upon complementation with *hupB* (Figure 6A).

The mycolic acid layer is a formidable barrier (Dover et al., 2004; Gebhardt et al., 2007) that protects Mtb from detergent-mediated damage and cell death. Several polyketide synthases including *ppsA, ppsC*, and *ppsE* play a key role in its biosynthesis, especially PDIMs (Goude and Parish, 2008; Bisson et al., 2012; Rens et al., 2021). Given the sensitivity of KO to even 0.05% SDS (Figure 6A), we next compared its *ppsA, ppsC* and *ppsE* transcript levels to those WT (Figure 6B). As expected, compared to the WT, the expression of all the three enzymes in the KO were significantly downregulated (Figure 6B). Given this observation, to evaluate if this downregulation of polyketide synthases in the KO influenced the levels/synthesis of different mycobacterial lipids, we performed detailed TLC lipid analyses (Chauhan et al., 2013; Sambandan et al., 2013) and compared the levels of different polar and apolar lipids of the KO and KO+*hupB* to that of the WT (Figure 6C). Interestingly, among the polar lipids, compared to the WT, the phospholipids (Ps) and diacylated-phosphatidyl-myo-inositol mannosides (AC_2_PIM_5_) of the KO were significantly reduced, while phosphatidylethanolamine (PE) was significantly enhanced (panel (i) of Figure 6C). As expected, in KO+*hupB*, most of the modulated lipids were restored and comparable to those of the WT (panel (i) of Figure 6C). Among the apolar lipids, PDIM-A and C (panel (ii) of Figure 6C) and α, methoxy and keto MAMEs (panel (iii) of Figure 6C) were significantly reduced in the KO. As expected, in KO+*hupB*, we observed a restoration of most lipids to WT levels (panels (i, ii, and iii) of Figure 6C). It is important to note that for superior visualization and comparative analyses of detectable levels of apolar lipids of the KO, we had to spot thrice the quantity of the one used for spotting the WT and KO+*hupB* (panels (ii and iii) of Figure 6C).

Since loss of *hupB* led to downregulated expression of type I polyketide synthases (Figure 6B), we wondered if *drrA* (Rv2936), an efflux pump present just adjacent to *ppsE*, is also downregulated. It is well-established that while *ppsA*-E is involved in the synthesis of several lipids including PDIMs (Trivedi et al., 2005; Goude and Parish, 2008; Bisson et al., 2012; Rens et al., 2021), *drrA*-C is involved in generation of an ABC transporter that also transports PDIMs to the required location on the surface of pathogenic mycobacteria (Remm et al., 2022). Interestingly, *drrA* levels were also significantly downregulated in the KO but not in the WT (Figure 6D). Finally, since the KO is susceptible to low amounts of INH, we tested if *katG*, the catalase peroxidase (Rv1908c) enzyme that converts INH to its active form (Ando et al., 2011), is upregulated. Surprisingly, we found that *katG* expression was marginally downregulated and not upregulated (Figure 6D).

## Discussion

Globally, directly observed treatment, short-course (DOTS) therapy has saved the lives of millions of patients with TB (Out, 2013; Mandal et al., 2017). Despite that, since the anti-TB drug cocktail induces several adverse effects, 1/3 to 1/2 of all patients with TB fail to diligently complete their recommended treatment regimen (Cazabon et al., 2017; Kaul et al., 2019). Such poor compliance has led to rapid emergence of drug-resistant mycobacteria and extended durations of treatment especially with second-line antibiotics that induce more severe adverse effects (Madhav et al., 2015; Kaul et al., 2019). Some of the side effects often generate a sequel and last lifelong. Consequently, this has warranted discovery of novel drug targets and anti-TB drugs that not only exhibit superior killing of infecting mycobacteria and reduced side effects but also help to reduce the daily dose intake of the existing drugs (Bhat et al., 2018; Shetye et al., 2020). We predict that our *in vitro* results with HupB, a prominent Mtb-encoded NAP, are broadly aligned to this effort.

Since NAPs help bacteria cope with stress, they are known to significantly accumulate *in vitro* during the mid to late log and stationary phases of growth (Bhat et al., 2018; Hołówka and Zakrzewska-Czerwińska, 2020). For example, IHF, MDP1, and Lsr2 accumulate in the stationary phase of growth (Ali Azam et al., 1999; Matsumoto et al., 2000; Kołodziej et al., 2021). Interestingly, we also find HupB accumulating in the mid to late log and stationary phases of Mtb growth (Figures 1A,B), indicating its possible role during stress. Global transcriptome datasets from different labs indicate that upon exposure to stress such as first-line drugs, especially INH, *hupB* expression levels are significantly increased (Reddy et al., 2009; Whiteford et al., 2011; Hadizadeh Tasbiti et al., 2016; Sakatos et al., 2018; Arora et al., 2021). A similar enhanced expression of *hupB* occurs in response to non-replicating persistence (Lee et al., 1998; Betts et al., 2002) and low-iron conditions (Pandey et al., 2014a). Interestingly, such increased expression also occurs in Msm with *hupB*'s ortholog *hlp* when Msm is exposed to different environmental stress conditions including anaerobic-induced dormancy (Lee et al., 1998; Shires and Steyn, 2001; Anuchin et al., 2010). Our semi-quantitative Western blot analyses of HupB protein levels during host-mediated and antibiotic-induced stresses align with these reports and demonstrate that HupB levels are indeed modulated in response to the stresses tested (Figure 1). Upon exposure to H_2_O_2_ and INH, HupB protein levels significantly increased (Figures 1C,E) and upon exposure to RIF, nitrosative, acidic, and nutrient stresses, HupB protein levels decreased (Figures 1C,D).

When compared to significant modulations of HupB protein levels during different phases of growth (Figures 1A,B) and stresses (Figures 1C–E), GroEL2 protein levels largely remained unaltered except that they significantly decreased during acidic stress (Figures 1C,D) and marginally decreased in the presence of RIF (Figure 1E). Given that RIF suppresses DNA-dependent RNA polymerase activity (Zhang et al., 2019), we did anticipate reduced accumulation of GroEL2 in Mtb in its presence (Figure 1E). The expression of *groEL2* is modulated when Mtb is present in macrophages (Monahan et al., 2001; Lin et al., 2016; Salina et al., 2019) and perhaps acidic stress is the main driver (Figures 1C,D). We infer that the reduced GroEL2 levels under nitrosative stress is primarily due to altered medium pH (from 7.4 to 5.2, Figure 1C).

Since HupB (i) aligns with several typical NAP properties; (ii) accumulates in late phases of growth *in vitro*, (iii) gets modulated during different stresses; and (iv) is an ortholog to Hlp whose KO is more sensitive to UV, cold shock, and exposure to INH (Shires and Steyn, 2001; Katsube et al., 2007; Mukherjee et al., 2009; Whiteford et al., 2011), we hypothesized that HupB also plays a definitive role in Mtb's response to host-mediated and antibiotic-induced stresses. This would imply that a *hupB* KO would exhibit increased sensitivity and susceptibility to these stresses.

Previously, a transposon insertion mutant of *hupB* was shown to restrict Mtb growth *in vitro* (Sassetti et al., 2003). As expected, our KO also exhibits a severely restricted growth phenotype *in vitro* (Figure 2). Growth retardation was more severe on the agar plates (Figure 2B) than in the axenic broth cultures (Figure 2A). The marked influence of HupB on *in vitro* growth, especially in low-iron conditions, has also been reported earlier (Pandey et al., 2014a). Furthermore, the KO also fails to survive in THP-1 (Figure 3G) and murine (Pandey et al., 2014a) macrophages, indicating HupB's role in Mtb growth in macrophages. Consistent with Pandey et al. (2014a), we also observed fewer KO bacteria able to infect and enter into THP-1 macrophages (Figure 3G). It is well-established that macrophages, besides generating acidic, nitrosative, and oxidative assaults, restrict Mtb growth by sequestering iron away from Mtb (Neyrolles et al., 2015; Sritharan, 2016; Upadhyay et al., 2018). It was earlier reported that the availability of siderophores is severely restricted in KO because, in WT, HupB binds to *hupB* boxes present upstream of mbt genes (Pandey et al., 2014a). Our comparative lipid profiling of the WT and KO (Figure 6C) indicates that perhaps the altered levels/almost loss of several polar and apolar lipids including PIMs, PDIMs, and mycolic acid esters in the KO influences its cell wall assembly and its architecture such that it significantly contributes to KO susceptibility to macrophage assaults and its inability to establish infection. Interestingly, the HupB ortholog Hlp also influences cell wall assembly in Msm (Katsube et al., 2007).

It is well-established that lipid composition and availability of lipids including PIMs and PDIMs significantly determine infection and virulence (Domenech and Reed, 2009). Since our KO contains reduced amounts of PDIM-A and –C, we evaluated the expression of three key type I polyketide synthases. Enzymes *ppsA* and *ppsC* are primarily involved in biosynthesis of the phthiocerol backbone of PDIM, while *ppsE* adds a methylmalonyl-CoA to phthiocerol (Trivedi et al., 2005; Bisson et al., 2012). Interestingly, when compared to the WT, the expression of all the three polyketide synthases in the KO was significantly reduced (Figure 6B). We predict that this altered lipid profile in the KO also largely influences its susceptibility to acidic (Figures 3A–C), oxidative (Figure 3E), nitrosative (Figure 3F), and nutrient depletion (Figure 3D). Reduced quantity of PDIMs and PIMs has been shown to increase cell wall permeability to detergents (Camacho et al., 2001), perhaps explaining why our KO exhibits altered cell wall permeability and enhanced susceptibility to 0.05% SDS (Figure 6A).

Since reduced levels of *ppsA*, C, and E expression led to reduced levels of PDIMs, we also checked the expression of the gene *drrA* that is located adjacent to *ppsE*. The gene *drrA* encodes for a nucleotide-binding domain of a type II ABC transporter, DrrABC (Remm et al., 2022), that transports PDIMs across the inner membrane for PDIM localization to Mtb's surface (Braibant et al., 2000; Remm et al., 2022). Gene *drrA* expression is very high in INH and RIF-resistant MDR and XDR Mtb isolates (Li et al., 2015; Coll et al., 2018; Khosravi et al., 2019), indicating its possible role as a drug efflux pump in increasing Mtb tolerance to INH, RIF, and other antibiotics (Remm et al., 2022). Our KO, when compared to the WT, shows barely any expression of *drrA* (Figure 6D) and, as expected, is highly susceptible to both INH and RIF (Figure 4, Supplementary Figures 6A, 7B). Upon exposure to different concentrations (i.e., folds MIC) of INH, although both the KO and the WT died within 7 days, the KO was highly susceptible to INH (Figure 4A). When compared to the WT, it requires a ~2,000-fold less amount of INH (Figure 4A). Further analyses indicates that the KO is not only susceptible to 1/5^th^ the MIC for INH (Figure 4A), but that it is also susceptible to this amount within 5 days (Supplementary Figure 6).

Interestingly, when compared to the logarithmic growth phase, the expression of the catalase gene *katG* during the stationary phase of growth is less; thus, Mtb cells that exhibit a lower amount of KatG become more tolerant to INH (Ando et al., 2011; Niki et al., 2012). Since lower quantity of INH kills KO but not WT bacteria (Figure 4A), we predicted that this phenotype may be due to *drrA* down regulation. Since *drrA* expression indeed goes down (Figure 6D), we then wondered if, in KO, *katG* expression also gets modulated or remains similar to that observed in WT. It is well-established that sensitivity to INH differences exists among the Mtb complex, *Mycobacterium marinum*, and non-tuberculous mycobacteria (NTM) primarily because of genetic variants of *katG*, and they determine the amount of INH required to kill different mycobacteria (Reingewertz et al., 2020). It turns out that the KO, when compared to the WT, exhibits a slightly reduced expression of *katG* (~20–40%, Figure 6D), and that seems to not influence KO's enhanced susceptibility to INH (Figure 4A).

The KO also exhibits enhanced susceptibility to RIF (Figure 4B). Upon exposure to different concentrations (i.e., folds MIC) of RIF, not only all KO bacteria died within 5 days (Supplementary Figure 7), they also required only a 10-fold lower amount of RIF than that required to kill all WT bacteria (Figure 4B). Importantly, to be killed, the WT requires higher amounts of RIF with longer duration of exposure (Figure 4B), thus indicating that enhanced levels of *hupB* make Mtb more tolerant to both RIF and INH. We also observed that overexpressing *hupB* enhanced WT's MIC to INH by 4-fold (Table 1, Supplementary Figure 9A) and to RIF by approximately a fold (Supplementary Figures 7, 10). Interestingly, *rpoB* mutants also modify Mtb into RIF-resistant bacteria, and these exhibit enhanced levels of PDIM and higher expression of *ppsA*-E (Bisson et al., 2012). In contrast, the *hupB* KO mutant not only accumulates significantly reduced amounts of PDIM-A and PDIM-C (Figure 6C) but also exhibits lower expression of *ppsA*, C, E (Figure 6B) and *drrA* (Figure 6D) and becomes more susceptible to lower concentration of RIF (Figure 4B). Despite the KO showing reduced levels of apolar lipids such as PDIMs and MAMEs (panels (ii) and (iii) of Figure 6C, respectively), surprisingly, the KO did not exhibit any enhanced sensitivity to the hydrophilic drug EMB (Supplementary Figure 8).

Our data (Figures 3, 4 and Table 1), albeit *in vitro*, clearly demonstrate that while the lack of *hupB* makes Mtb more susceptible to stresses and antibiotics, the presence and enhanced accumulation of HupB makes Mtb cope with them. Specifically, since lack of *hupB* makes Mtb susceptible to significantly low quantity of INH, when we inhibited WT HupB with a stilbene inhibitor, *viz*., SD1, and then tested the sensitivity of the SD1-treated WT strain to INH, excitingly, the combination of SD1 and INH, despite not being synergistic (Figure 5A), increased the efficacy of SD1 by 2-fold and INH by 4-fold (Figure 5A, Supplementary Table 5) and rapidly killed the axenically cultured bacteria with just 4× MIC of INH (Figure 5B) as compared to the 400 × MIC of INH required for the WT (Figure 4A). As expected, we could also significantly kill more THP-1 macrophage-infecting WT bacteria upon combinatorial treatment with SD1 and INH as compared to SD1 and INH alone (Figure 5C), again indicating that targeting HupB helps significantly reduce the dose of INH necessary to kill Mtb.

Although one desires and strives hard to discover an alternate, superior, and novel therapeutic target that supplants the current anti-TB therapeutic molecules, we predict that achieving such a goal is extremely challenging. As an alternate and potentially achievable target that may better support the United Nations (UN) Sustainable Development Goals and is aligned with the UN combinatorial therapy recommendations, we speculate that HupB-targeting together with INH and RIF holds a great promise. While we have just begun our efforts to pre-clinically recapitulate our *in vitro* observations with HupB, in principle, we emphasize that HupB is a very promising combinatorial target when used together with RIF and INH. Given the high MIC for SD1 (Supplementary Table 4), we clearly echo that an alternate to SD1 is absolutely essential to take this target to the next level of promising ones. One promising candidate is the small molecule “3d” developed very recently (Peraman et al., 2021). We are also currently screening a library of small molecules for an alternate to SD1 and “3d”.

In summary, our detailed *in vitro* studies with *hupB* and its significant role in helping Mtb cope with stress suggest a model wherein targeting Mtb's HupB with a small molecule inhibitor helps to significantly reduce the expression of type-I polyketide synthases. This, in turn, significantly reduces the levels of several lipids including virulent PDIMs. This promotes increased permeability of the pathogen membrane and enhanced susceptibility to host-mediated stresses (in macrophages) and antibiotic-induced stresses (during treatment). The simultaneous reduction in the expression of PDIMs synthesizing enzymes and *drrA* (and perhaps *drrB* and C because together they form one transporter and are genetically organized as an operon) perhaps prevents any efflux/expulsion of administered INH and RIF, thus significantly reducing their MICs and duration of therapeutic treatment.

## Data availability statement

The original contributions presented in the study are included in the article/Supplementary material, further inquiries can be directed to the corresponding author/s.

## Author contributions

KA: conceptualization, methodology, resources, supervision, project administration, funding acquisition, data analysis, writing review and editing, and final revision. RS: methodology, resources, and supervision. NSi, NSh, PS, MP, MI, LS, TC, and TG: investigation and data generation. KA, RS, NSi, and PS: formal analysis. KA, NSi, PS, MP, and MI: writing original draft preparation. All authors have read and agreed to the published version of the manuscript.

## Funding

This research was primarily funded by the Translational Health Science and Technology Institute's internal grant to KA supported through the Department of Biotechnology, India. KA is a recipient of a Ramalingaswami fellowship from the Department of Biotechnology, India. The Department of Biotechnology, India, funded NSi through a graduate program fellowship. RS acknowledges the funding received from the Department of Biotechnology, India (grant ID BT/PR30215/MED/29/1343/2018). RS is also a recipient of a Ramalingaswami fellowship and National Bioscience Award from the Department of Biotechnology, India, and is a senior fellow of Wellcome Trust-DBT India Alliance (IA/S19/2/504646), India. PS sincerely acknowledges the Science and Engineering Research Board, Government of India for his national post-doctoral fellowship.

## Conflict of interest

The authors declare that the research was conducted in the absence of any commercial or financial relationships that could be construed as a potential conflict of interest.

## Publisher's note

All claims expressed in this article are solely those of the authors and do not necessarily represent those of their affiliated organizations, or those of the publisher, the editors and the reviewers. Any product that may be evaluated in this article, or claim that may be made by its manufacturer, is not guaranteed or endorsed by the publisher.
