# Supplementary material for: HupB, a nucleoid-associated protein, is critical for survival of Mycobacterium tuberculosis under host-mediated stresses and for enhanced tolerance to key first-line antibiotics
Source: Front Microbiol. 2022 Aug 22;13:937970. doi: 10.3389/fmicb.2022.937970 (PMC9441915; doi:10.3389/fmicb.2022.937970)
Supplement: Supplementary file 1 [file Data_Sheet_1.pdf]

Revised Supplementary Materials

Manuscript ID: 937970

Title: HupB, a nucleoid-associated protein is critical for survival of *Mycobacterium tuberculosis* under host-mediated stresses and for enhanced tolerance to key first-line antibiotics

Fig. S1: HupB protein levels significantly alter during different phases of Mtb growth (in rich and minimal/nutrient depleted media) *in vitro.*

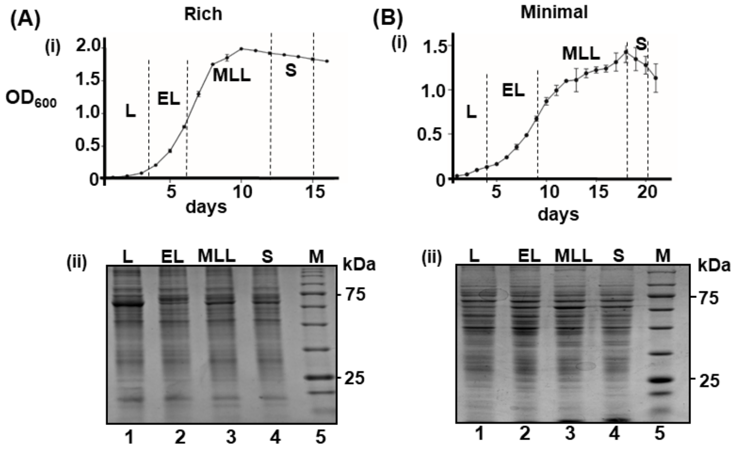

<u>**Legend:**</u> Mtb (H37Rv; WT) was grown to different phases (L, Lag; EL, Early log; MLL, Mid to Late log; and S, Stationary – demarcated with dashed lines) in rich **(A)** and nutrient-depleted/minimal (**B**) media. **Panels (i) of A & B:** are representative growth curve plots with optical densities (recorded as technical duplicates) of biological triplicate cultures (OD_{600} (optical density at A_{600nm})) plotted (Y-axis) against days of incubation (X-axis). Technical duplicate OD_{600} readings were recorded and averaged, SD values calculated and growth curves plotted. Panels **(ii)** of **A** & **B:** Bacteria grown to different phases of growth (as shown in panels **(i)** of **A** & **B**)) were pelleted down (to represent the phases indicated), washed, bead beaten and lysed. Equal quantity of total lysate proteins (~30 µg) were boiled for 15 min in 1X Laemmli's sample buffer, resolved (in 15% SDS-PAGE) and coomassie stained (the black & white image is shared here) for visualization of denatured proteins. Two gels were simultaneously run in parallel. While one was used for western analyses (panels **(i)** of Fig. **1A & B**), the other was stained with coomassie to ensure loading of equal quantity of protein. kDa, kilo Daltons; M, reference protein marker; (BioRad (Cat # 1610373)); Numericals underneath the coomassie gel images (panels **(ii)** of **A & B**) indicate gel lane numbers.

Fig. S2: HupB protein levels significantly alter in response to Mtb exposure to different host-mediated stresses imposed *in vitro.*

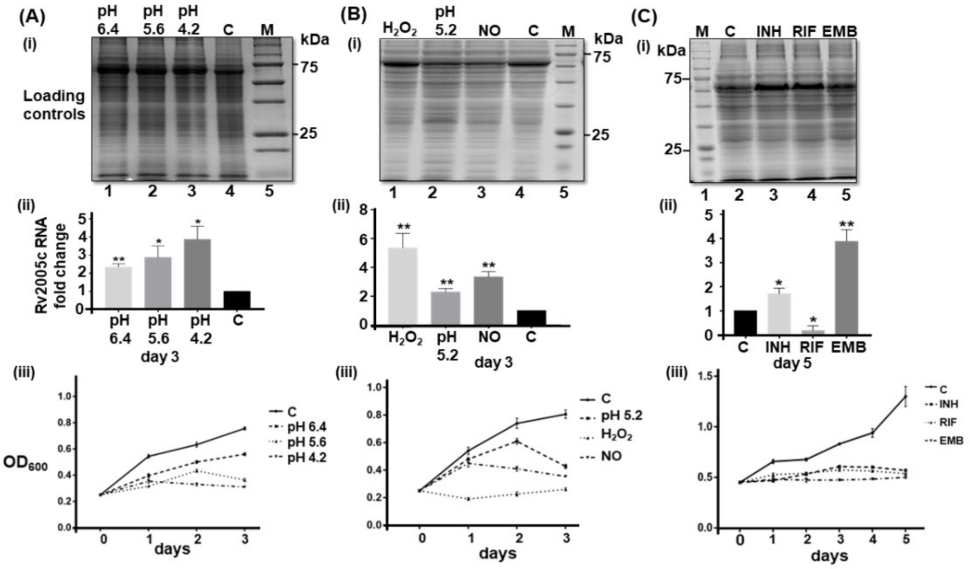

<u>**Legend:**</u> WT grown in rich media to ~0.8 OD_{600} was washed and sub cultured at 0.05 OD_{600}. When absorbance reached ~0.2-0.3 OD_{600} (for **A** & **B**) or ~0.4-0.6 OD_{600} (for **C**), all cultures were washed and normalized to 0.25 OD_{600} (for **A** & **B**) or 0.4 OD_{600} (for **C**). Around 50 mL cultures were then imparted with different stresses. (**A-C**): Bacteria from all cultures were pelleted down, washed, bead beaten and lysed. Equal quantity of lysate proteins (~30 µg) was boiled for 15 min in 1X Laemmli's sample buffer, resolved (in 15% SDS-PAGE) and coomassie stained (black & white image shown here; panels **(i)** of **A-C**). Two gels were simultaneously run in parallel - one for western analyses (**panels (i) of Fig. 1**) while the other was stained with coomassie to ensure loading of equal quantity of total proteins. **Panels (ii) of A-C**: Fold change of Rv2005c (a universal stress protein - **Hingley-Wilson et al., (2010))** was plotted (*sigA* used as house-keeping reference control) to indicate stress imposition. Total RNA was extracted from ~10^9 bacteria, quantitated, equal quantity RNA treated with TURBO DNase, cDNA synthesized and RT-PCR performed (details in Materials and Methods). **A**: pH stress (pH 6.4, 5.6 and 4.2) was imposed for 3 days (d) in rich media). **B**: H_2O_2, oxidative stress was imposed for 3 d with 5 mM Hydrogen peroxide; NO, nitrosative stress was imposed for 3 d with 1 mM Sodium nitrite in pH 5.2 (rich media). **C:** INH, Isoniazid (2.91 µM); RIF, Rifampicin (6 nM); EMB, Ethambutol (9.79 µM) – all antibiotics exposures were for 5 d. kDa, kilo Daltons; **C** (in panels) indicate control (no treatment, bacteria grown in rich media); M, protein marker; (Genedirex protein ladder (Cat # PM007-0500) used for blot **A-i** & BioRad (Cat # 1610373) used for **B-i and C-i**). **Panels (iii) of A-C:** Representative growth curves of WT under different conditions of stress with OD_{600} (optical density at A_{600nm})) plotted (Y-axis) against days of incubation (X-axis).

Numericals underneath coomassie stained gel images indicate gel lanes. Rv2005c fold change was determined using biological triplicates and technical duplicates and control plotted as fold change value "1". Student t-test was performed for comparative analyses with control and significance evaluated. *p <0.05; **p <0.01; ns non-significant. The data are representative of biological triplicates.

Reference: Hingley-Wilson, S.M.; Lougheed, K.E.A.; Ferguson, K.; Leiva, S.; Williams, H.D. Individual Mycobacterium Tuberculosis Universal Stress Protein Homologues Are Dispensable in Vitro. *Tuberc. Edinb. Scotl.* 2010, *90*, 236–244, doi: 10.1016/j.tube.2010.03.013.

Fig. S3: Construction, confirmation and complementation of *hupB* KO (WT∆*hupB*) mutant.

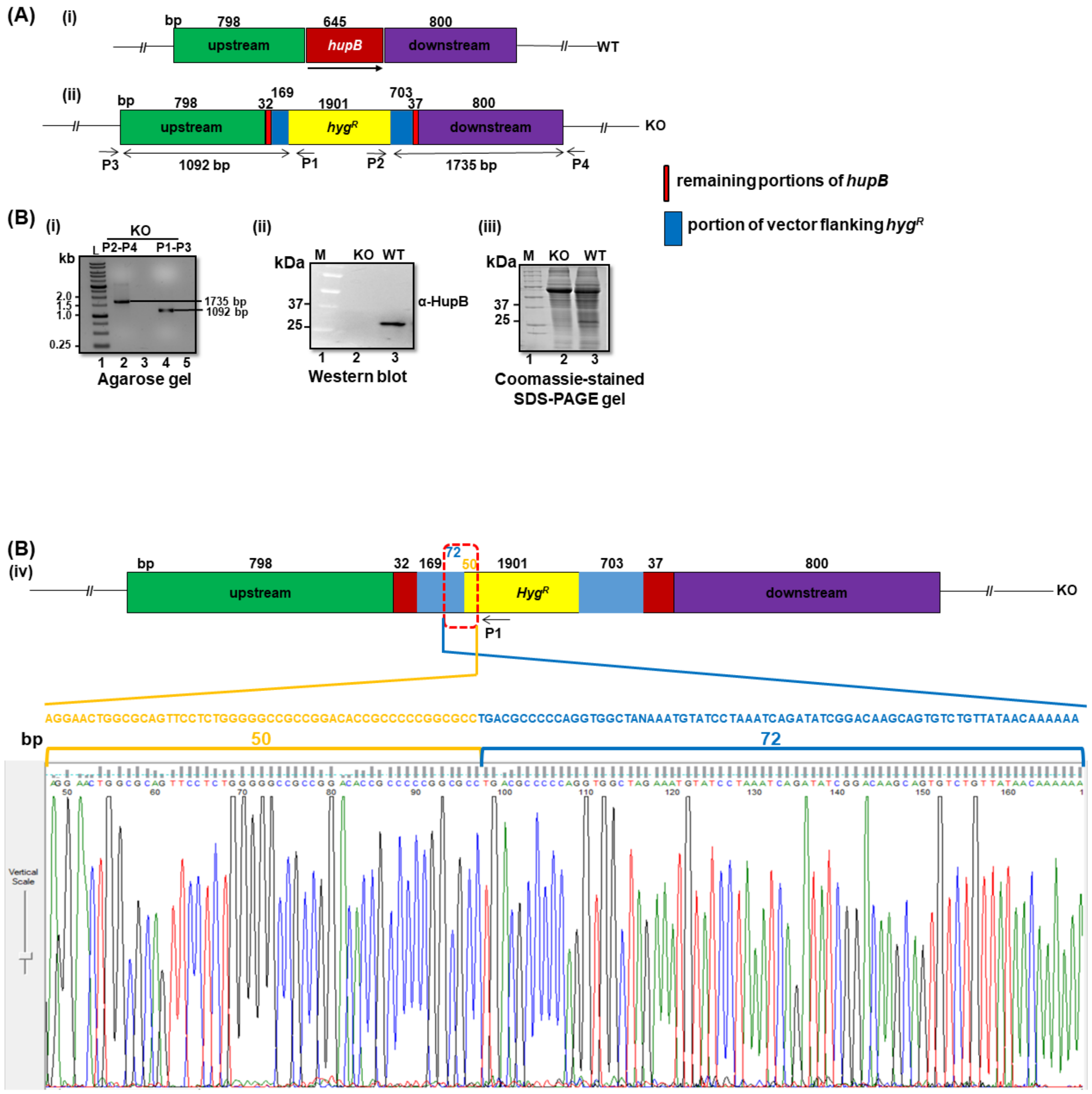

Junction sequence (50 bp of *hyg*^{R} + 72 bp of **vector** (used for moving Hyg^R into *hupB*; sky blue) generated with reverse primer, P1 (Table S3).

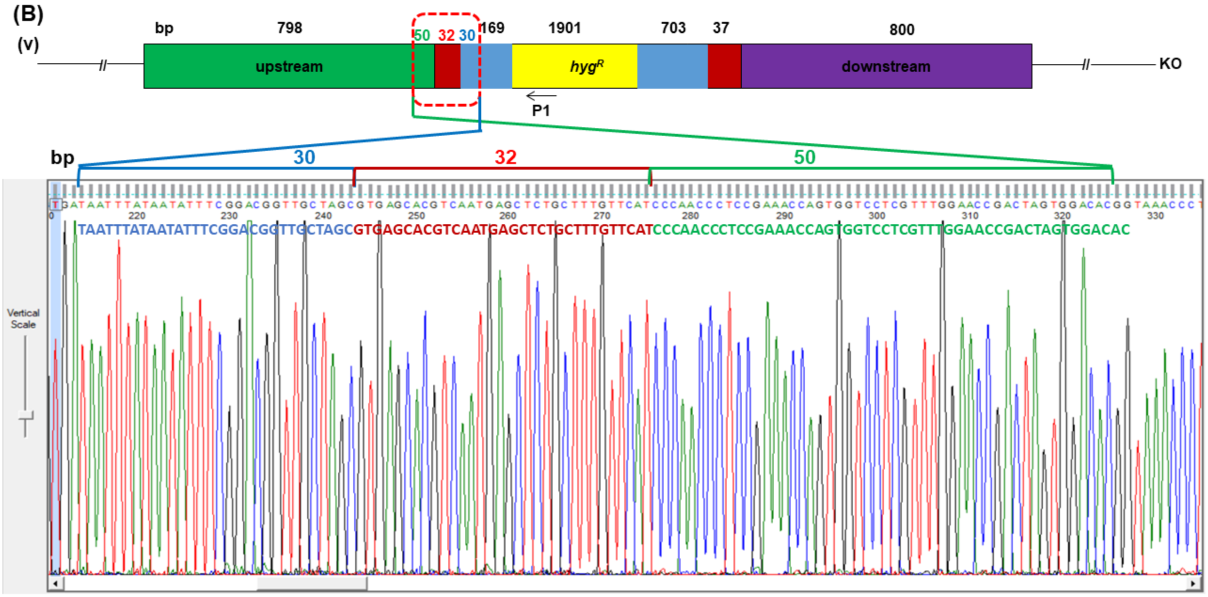

Junction sequence (30 bp of **vector** (used for moving *hyg*^{R} into *hupB*) + 32 bp of ***5'-hupB*** + 50 bp of **upstream**, generated with reverse primer, P1 (Table S3).

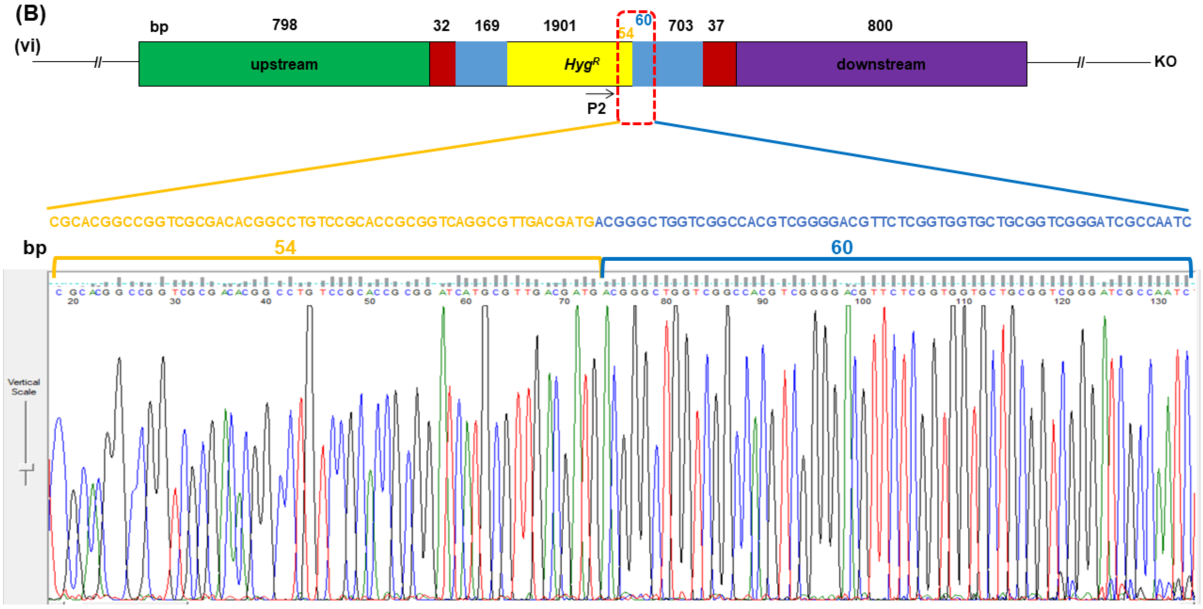

Junction sequence (54 bp of ***hyg***^{R}) + 60 bp of of **vector** (used for moving *hyg*^{R} into *hupB*), generated with forward primer, P2 (Table S3).

(B)

(vii)

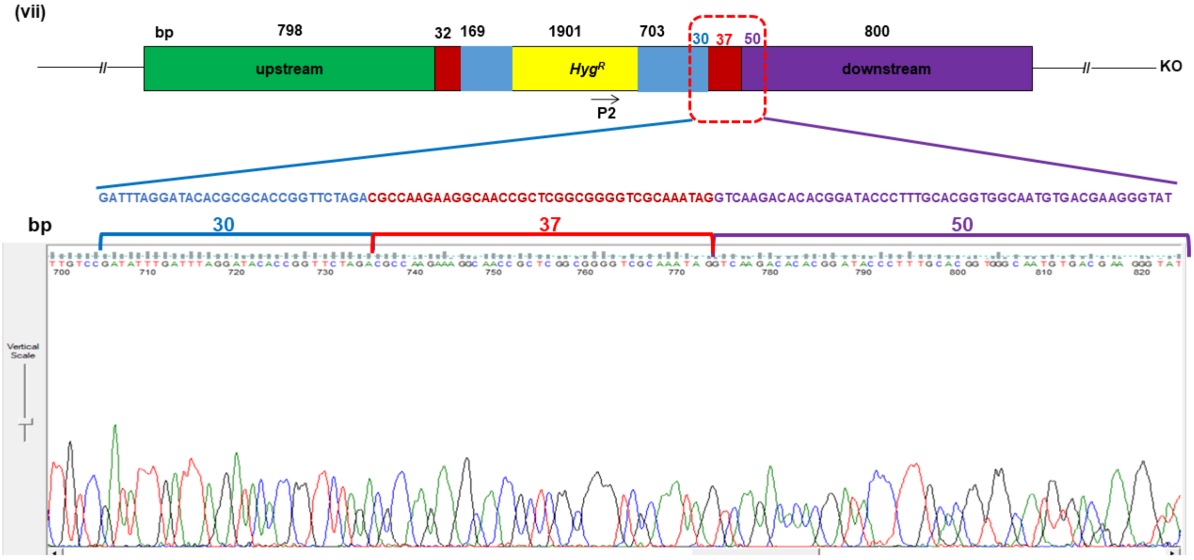

Junction sequence (30 bp of **vector** (used for moving *hyg*^{R} into *hupB*) + 37 bp of ***3'-hupB*** + 50 bp of **downstream**, generated with forward primer, P2 (Table S3).

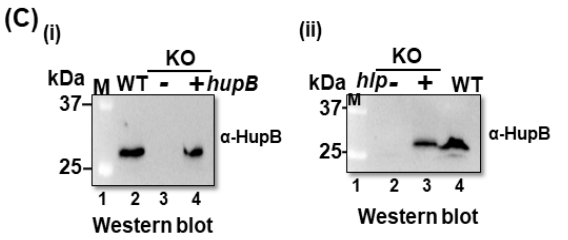

Legend: **(A):** Construction of KO: Schematic representation of *hupB* locus in WT **(i)** and in KO **(ii)** before and after double crossover homologous recombination and insertion of Hygromycin resistant cassette (*hyg*^{R}) (Note: a small portion of the vector (used for recombination) that flanks the cassette is also inserted after double crossover). Single straight line with line-breaks that extend to the left and right of rectangular blocks (green and purple respectively) represent rest of the genome that is upstream and downstream to *hupB* locus (arrow indicates direction of *hupB* ORF). Upstream: immediate upstream region to *hupB* (798 bp; green), downstream: immediate downstream region to *hupB* (800 bp; purple). Length of each rectangular block (not drawn to scale) is indicated in base pairs (bp) on the top. P1 and P2: internal (reverse and forward respectively) primers at the 5' and 3' ends of Hygromycin resistant cassette; P3, forward primer immediately upstream of 798 bp upstream region; P4, reverse primer immediately downstream of 800 bp downstream region; amplicons length (in bp) with P1 & P3 and P2 and P4 sets of primers are indicated at the bottom of **(ii)**. **(B):** Confirmation of KO: 0.8% agarose gel image **(i)** exhibiting amplicons (in bp) with P2 & P4 (lane 2) and P1 & P3 primers (lane 4). L, 1 kb plus ladder, lanes 3 and 5 are blank. WT and KO were grown to ~1.0 OD600 in rich media, cells pelleted down, washed, bead beaten and lysed. Equal quantity of protein (~ 30 µg) boiled for 15 minutes in 1X Laemmli's sample buffer were resolved in 15% SDS-PAGE and western (ii) and coomassie **(iii)** performed. Western analysis involved use of purified anti-HupB antibody. **(iv) to (vii):** Sanger sequencing of KO confirming insertion of Hygromycin resistant cassette (*hygR*) into *hupB* locus. Schematic representation is provided in each panel **(iv to vii)** followed by the original chromatogram data. For clarity, only the junction sequences are shown. **(C):** Complementation of KO: WT, KO and KO complemented with either *hupB* (from WT); **(i)**) or *hlp* (from Msm – mc^{2}155; **(ii)**) were grown to ~1.0 OD600 in rich media (30 mL), cells pelleted down, washed, bead beaten and lysed. Equal quantity of protein (~ 30 µg (for western) boiled for 15 minutes in 1X Laemmli's sample buffer were resolved on 15% SDS-PAGE and western performed with purified anti-HupB antibody.

Fig. S4: Loss of *hupB* makes Mtb highly susceptible *in vitro* to host-simulated stress conditions.

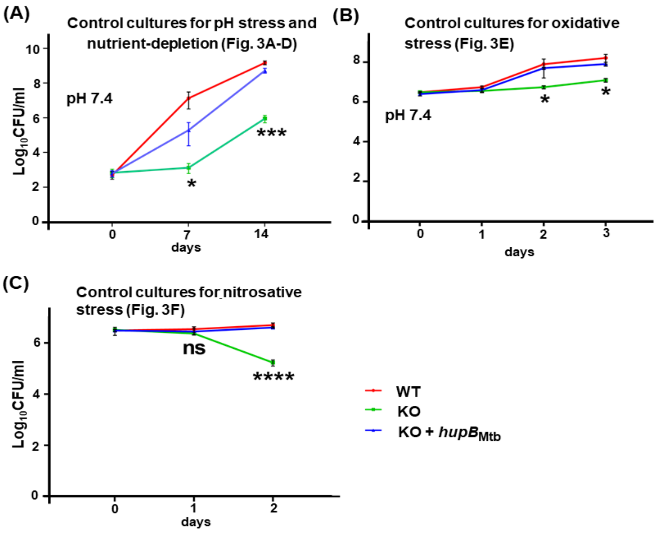

Legend: WT (red), KO (green) and KO+*hupB* (blue) were grown in rich media to ~0.8 OD_{600}, washed and sub-cultured at 0.05 OD_{600}. When absorbance reached ~0.2-0.3 OD_{600}, they were washed and cultures adjusted to 0.25 OD_{600}. Around 5 mL cultures each were imparted with pH, nutrient, oxidative, nitrosative, and nutrient-depleted stresses. **(A)** represents comparative growth patterns of WT, KO and KO+*hupB* at pH 7.4 (represent as control cultures for **Fig. 3A-D**); **(B)** represent growth progression of control cultures of WT, KO and KO+*hupB* at pH 7.4 (portion of primary cultures processed and subject to oxidative stress (**Fig. 3E**). Similarly, **(C)** represents growth progression of control cultures of WT, KO and KO+*hupB* at pH 5.2, (portion subjected to nitrosative stress with sodium nitrite (**Fig. 3F**). At specified time points (X-axis), cultures were plated directly on appropriate media and Colony Forming Units (CFUs) enumerated. The numbers obtained were averaged, SD value calculated and then plotted as log_{10}CFUs/mL scale on the Y-axis. Similarly, Student t-test was performed for comparative analyses and significance evaluated. *p <0.05; ** p <0.01; *** p<0.005; **** p<0.001; ns non-significant. The data is a representation of biological triplicates and technical duplicates.

Fig. S5: Minimum Inhibitory Concentration (MIC) *in vitro* to INH in Mtb as determined by alamarBlue assay.

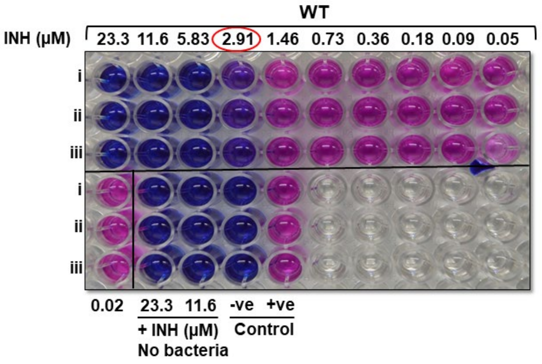

Legend: WT was grown in rich media to ~0.4 – 0.6 OD_{600}, washed and then diluted to 0.0015 OD_{600}. Approx. ~ 3.75 X 10^4 CFUs was exposed to 2 fold increments of INH (μM; labelled above and below 96 well plate) for 5 d. Required volume of alamarBlue (to final 1X) was added and incubated at 37°C incubator for an additional 48 h and blue to pink color change recorded. i, ii, iii to the left indicates biological triplicate cultures. Drug only controls (+ INH & No bacteria) include all components except bacteria. +ve control, ONLY bacteria (no drug control); -ve control, ONLY media (no drug and no bacteria control). The red oval circling the indicated concentration represents the MIC under our conditions. **NOTE:** the bacterium harbors a plasmid vector (pVV16), used as control strain for WT over expressing *hupB* (Fig. S9).

Fig. S6: Loss of *hupB* makes Mtb highly susceptible *in vitro* to reduced amount of INH.

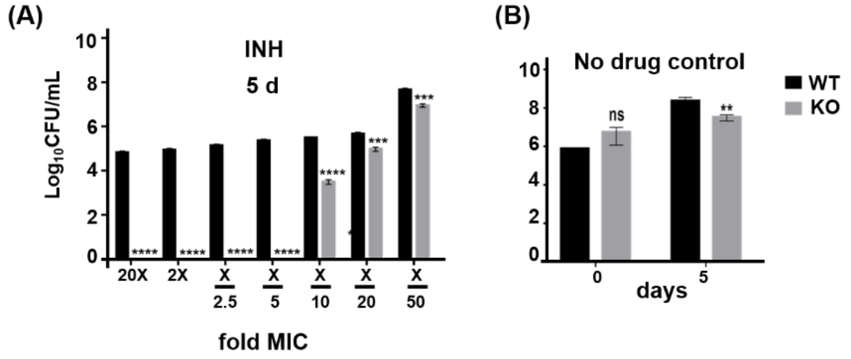

Legend: WT (black bars) and KO (grey bars), were separately grown *in vitro* in rich media to ~0.8 OD_{600}, washed and sub cultured at 0.05 OD_{600}. When absorbance reached 0.4-0.6 OD_{600}, they were washed, then normalized to 0.1 OD_{600}. Around 5 mL cultures were imparted stress with INH **(A)** for 5 d at different folds MIC (X-axis; MIC denoted as X (2.91 µM) and survivors enumerated (as CFUs), averaged, SD value calculated and then plotted (Log_{10}CFUs/mL, Y-axis)**.** Student t-test was performed for comparative analyses and significance evaluation. ** p <0.01; ***p <0.005; ****p <0.001. ns – not significant. The data shown is a representation for biological triplicate cultures with technical duplicate platings. **(B)** No drug control (without exposure to antibiotics) plotted for comparative enumeration of WT and KO at 0 and 5 days. Note: **(B)** is identical image to Fig. S7C and S8C as all three experiments were performed simultaneously with same biological triplicate cultures.

Fig. S7: Loss of *hupB* makes Mtb highly susceptible *in vitro* to reduced amount of RIF.

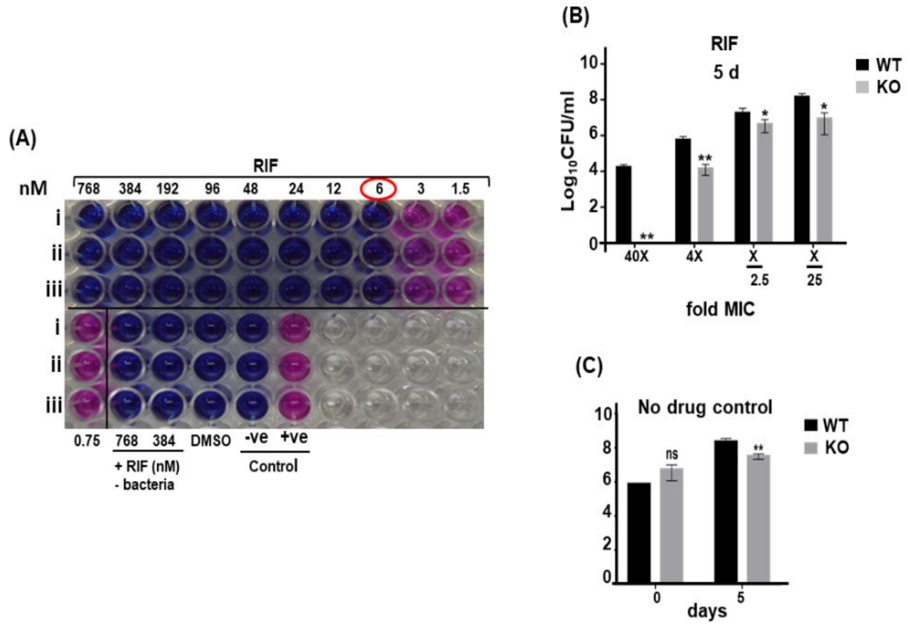

Legend: (A): Determination of MIC on Mtb to RIF. WT was grown in rich media to ~0.4-0.6 OD_{600}, washed and diluted to 0.0015 OD_{600}. Approx. 3.8 X 10^4 CFUs were then exposed to 2-fold increments of RIF (denoted as nM; labelled above and below 96 well plate) for 5 d. Required volume of alamarBlue (to final of 1X) was added, incubated at 37ºC for 48 h and color change recorded. i,ii,iii to the left of the 96 well image indicates biological triplicate cultures. Drug only controls (+ RIF & - bacteria) include all components except bacteria. +ve control, ONLY bacteria (no drug control); -ve control, ONLY media (no drug and no bacteria control). The red oval circling the indicated concentration represents the MIC under our conditions. **(B):** WT (black bars) and KO (grey bars), were separately grown *in vitro* in rich media to ~0.8 OD_{600}, washed and sub cultured at 0.05 OD_{600}. When absorbance reached 0.4-0.6 OD_{600}, they were washed, then normalized to 0.1 OD_{600}. Around 5 mL cultures were imparted stress with Rifampicin (RIF) for 5 d at different folds MIC (X-axis; MIC denoted as X) and survivors enumerated (as CFUs), averaged, SD value calculated and then plotted (Log_{10}CFUs/mL, Y-axis)**.** X for RIF is 6

nM (see panel A). Student t-test was performed for comparative analyses and significance evaluation. ** p <0.01; ***p <0.005; ****p <0.001. ns – not significant. The data shown is a representation for biological triplicate cultures with technical duplicate platings. **(C)** No drug control (without exposure to antibiotics) plotted for comparative enumeration of WT and KO at 0 and 5 d. Note: **(C)** is identical image to Fig. S6B and S8C as all three experiments were performed simultaneously with same biological triplicate cultures.

Fig. S8: Loss of *hupB* does not alter Mtb susceptibility *in vitro* to EMB.

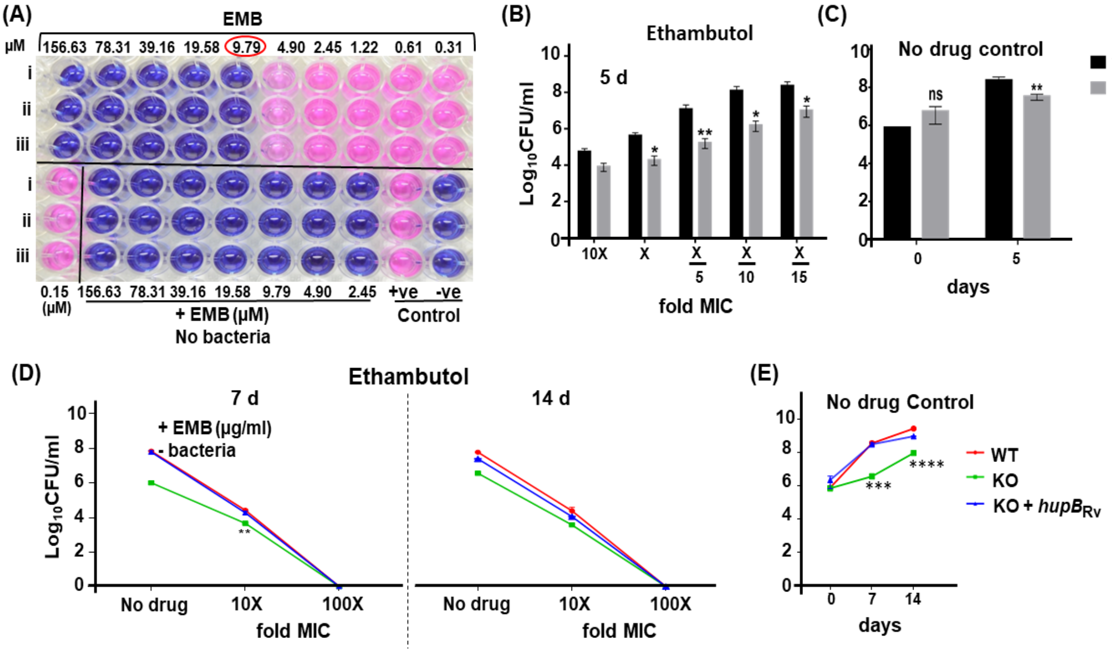

Legend: **(A)**: Determination of MIC for EMB on Mtb by alamarBlue Assay**.** WT was grown in rich media to ~0.4 -0.6 OD_{600}, washed and diluted to 0.0015 OD_{600}. Approx. 4.2 X 10^4 CFUs were then exposed to 2-fold increments of EMB (µM - denoted above and below the 96 well plate image) for 5 d. Required volume of alamarBlue (to final of 1X) was added, incubated at 37°C for 48 h and color change recorded. i, ii, and iii to the left of the 96 well image indicates biological triplicate cultures. Drug only controls (+ EMB & No bacteria) include all components except bacteria. +ve control, ONLY bacteria (no drug control); -ve control, ONLY media (no drug + no bacteria control). The red oval encircling the indicated concentration represents the MIC under our experimental conditions; **(B)**: WT (black) and KO (grey) were grown in rich media to ~0.4-0.6 OD_{600}, washed and subcultured to 0.1 OD_{600}. Around 5 mL of cultures were then exposed for 5 d to different folds MIC of EMB (µM; X-axis). Cultures were then plated on appropriate media and CFUs enumerated, averaged, SD value calculated and plotted as log_{10}CFUs/mL (Y-axis); **(C):** No drug control - CFUs of WT and KO of day 0 and 5 Note: **(C)** is identical image to Fig. S7C and S8C as all three experiments were performed simultaneously with same biological triplicate cultures; **(D):** WT (red), KO (green) and KO+*hupB* (blue) were grown *in vitro*, washed, sub cultured at 0.1 OD_{600}. Around 5 mL of cultures were then exposed for 7 and 14 d to 10X and 100X EMB. Cultures were plated on appropriate media at the time (days) denoted on X-axis and CFUs enumerated, averaged, SD value calculated and then plotted as log_{10} scale on the Y-axis. Student t-test was performed for comparative analyses and significance evaluated. *p <0.05; ** p <0.01. The data is a representation of biological triplicates and technical duplicates. **(E)** Growth (at 7 and 14 d) of the three mycobacterial strains used in **(D)** without any antibiotics.

Fig. S9: Overexpression of *hupB* in Mtb significantly enhances MIC *in vitro* to INH.

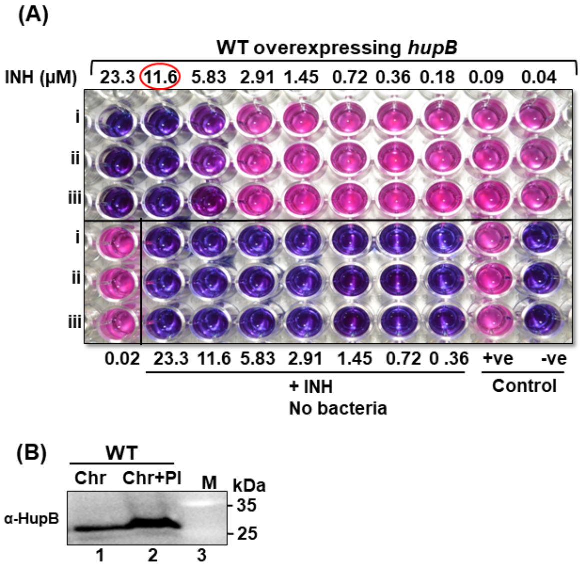

Legend: **(A):** WT with pVV16 + *hupB* was grown in rich media to ~0.4 – 0.6 OD_{600}, washed and diluted to 0.0015 OD_{600}. Approx. 3.75 X 10^4 CFUs was then exposed to 2 fold increments of INH (denoted as µM; labelled above and below 96 well plate) for 5 d. Required volume of alamarBlue (to final 1X) was added and incubated at 37ºC incubator for 48 h and color change recorded. i, ii, iii to the left indicates biological triplicate cultures. Drug only controls (+ INH & No bacteria) include all components except bacteria. +ve control, ONLY bacteria (no drug control); -ve control, ONLY media (no drug and no bacteria control). The red oval circling the indicated concentration represents the MIC under our conditions. (Compare results of MIC with data of Fig. S5).

(B): To observe for overexpression, WT with vector (plasmid for episomal-based expression) alone (pVV16) or with pVV16 + *hupB* were grown to 0.4 OD_{600}, pelleted down, washed and lysed. Equal quantity of protein (~ 30 µg) boiled for 15 minutes in 1X Laemmli's sample buffer was resolved on 15% SDS-PAGE and western performed with anti-HupB antibody. Chr, chromosome-based expression alone; Chr + Pl, Chromosome and plasmid-based expressions.

Fig. S10: Overexpression of *hupB* in Mtb marginally enhances MIC *in vitro* to RIF.

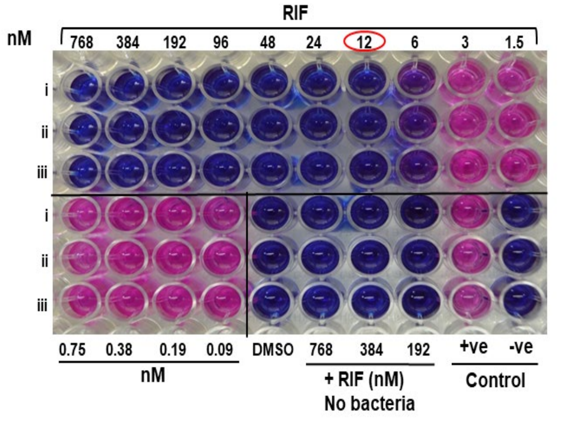

Legend: WT with pVV16+*hupB* was grown in rich media to ~0.4-0.6 OD_{600}, washed and diluted to 0.0015 OD_{600}. Around 3.75 X 10^4 CFUs were then exposed to 2-fold increments of RIF (denoted as nM; labelled above and below 96 well plates) for 5 d. Required volume of alamarBlue (to final 1X) was added and incubated at 37ºC

incubator for 48 h and color change recorded. i, ii, iii to the left indicates biological triplicate cultures. Drug only controls (i.e., + RIF & No bacteria) include all components except bacteria. +ve control, ONLY bacteria (no drug control); -ve control, ONLY media (no drug and no bacteria control). The red oval circling the indicated concentration represents the MIC under the overexpressed *hupB* conditions. (Compare results of MIC with data of Fig. S7)

Fig. S11: Overexpression of *hupB* in Mtb does not enhance MIC *in vitro* to EMB.

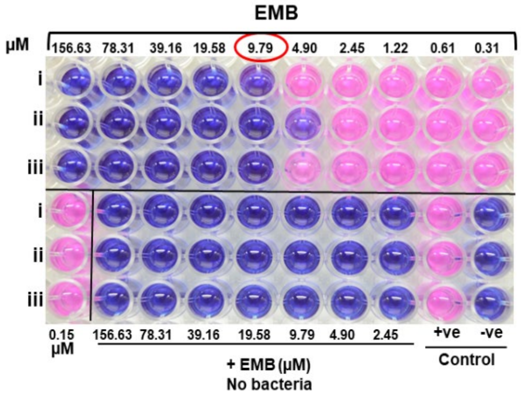

Legend: WT with pVV16+*hupB* was grown in rich media to ~0.4-0.6 OD_{600}, washed and diluted to 0.0015 OD_{600}. Around 4 X 10^4 CFUs were then exposed to 2-fold increments of EMB (denoted as μM; labelled above and below 96 well plates) for 5 d. Required volume of alamarBlue (to final 1X) was added and incubated at 37ºC incubator for 48 h and color change recorded. i, ii, iii to the left indicates biological triplicate cultures. Drug only controls (i.e., + EMB & No bacteria) include all components except bacteria. +ve control, ONLY bacteria (no drug control); -ve control, ONLY media (no drug and no bacteria control). The red oval circling the indicated concentration represents the MIC under the overexpressed *hupB* conditions. (Compare results of MIC with data of Fig. S8).

Fig. S12: SD1 and INH are not toxic to THP-1 macrophages at high concentrations

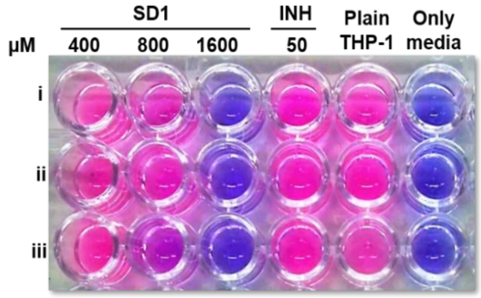

Legend: Around 10^5 THP-1 macrophages were seeded and PMA differentiated. Different concentrations of SD1 and 50 μM of INH were added into appropriately labelled wells (indicated on the top). They were incubated in CO_2 incubator for 48 h at 37°C. alamarBlue was added (to 1X final concentration) and color change recorded. i, ii, iii indicate biological triplicates.

Fig. S13: Specificity of Stilbene (SD1) to HupB.

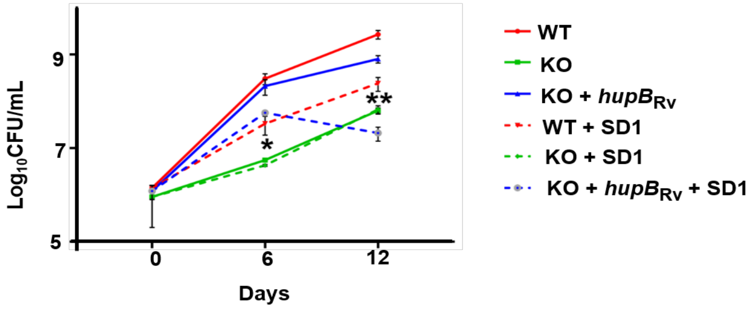

Legend: WT (red), KO (green) and KO+*hupB* (blue) were grown *in vitro* to ~0.3 OD_{600}, washed and sub-cultured to 5 mL cultures each of 0.1 OD_{600} and then treated with 100 µM of SD1 (dash lines) for days specified in X-axis. Bacteria were plated on appropriate media and numbers enumerated, averaged, SD value calculated and then plotted as log_{10}CFUs/mL (Y-axis). Student t-test was performed for comparative analyses and significance evaluated. *p <0.05; **p <0.01. The data is a representation of biological triplicates and technical duplicates

Table S1: Bacterial strains list

Bacterial strain	Details	Source
Escherichia coli DH5α	for cloning	Thermo Fisher Scientific
Escherichia coli BL21 pLysS DE3	for protein expression	Thermo Fisher Scientific
Escherichia coli HB101	for cloning	Thermo Fisher Scientific
Mycobacterium Smegmatis (mc^{2}155)	Avirulent mycobacterium	Kind gift from Prof. Sarah M. Fortune
Mycobacterium tuberculosis (H37Rv – referred to as WT)	Pathogenic mycobacterium	Kind gift from Prof. Sarah M. Fortune
WT harbouring pVV16+*hupB*	For constitutive overexpression of *hupB*	This study
KO (WT*ΔhupB*)	*hupB* knockout mutant derived from WT	This study
KO harbouring pVV16+*hupB*	KO complemented with *hupB*	This study
KO harbouring pVV16+*hlp*	KO complemented with *hlp*	This study

Table S2: Plasmids list

Plasmid	Details	Source
pVV16	Constitutively expression vector in WT	Yaseen *et al.*, 2018
pET28a	*E. coli* expression vector	Novagen
pYUB584	Cloning vector	Bardarov *et al.*, 2002
pNA1	pET28a+*hupB*	This study
pNA2	pYUB584+798 bp upstream of *hupB*	This study
pNA3	pNA2+800 bp downstream of *hupB*	This study
pNA4	pVV16+*hupB*	This study
pNA5	pVV16+*hlp*	This study
pNA6	pNA3+λ DNA	This study

Table S3: Primers list.

Primer	Details	Sequence	Reference
**KAP 370 F**<br>**KAP 371 R**	Upstream (P3)<br>Downstream (P4) of *hupB* (Fig. S3A(ii))	**5′ GTCGTGTACCAACGGTCGCATTGAAGATCTG 3′**<br>**5′ GGTGCTGGGAGCTCGGGCCCGACGGGCAG 3′**	This study
**KAP 403 F**<br>**KAP 404 R**	Cloning *hupB* for protein expression	**5´AGACTACATATGATGAACAAAGCAGAGCTCATTGACGTGCTC 3´**<br>**5´ACTACGGATCCCTATTTGCGACCCCGCCGAGCGGTTGCCTTC 3´**	This study
**KAP 451 F**<br>**KAP 452 R**	For real time PCR of *hupB*	**5' GCAGCCAAGAAGGTAGCGAAG 3'**<br>**5' CCGCCTTGGTCACCTTCTTG 3'**	This study
**KAP 461 F**<br>**KAP 462 R**	For real time PCR of Rv2005c	**5' GTCTCGCCGAACGCTTGG 3'**<br>**5' CTGACCGACCCCAGAAGC 3'**	This study
**KAP 475 F**<br>**KAP 476 R**	For cloning upstream of *hupB*	**5' GTCGAAGCTTTCACACCCACTCTGGTATTGGCG 3'**<br>**5'GACAGCTAGCGTGAGCACGTCAATGAGCTCTGCTT 3'**	This study
**KAP 477 F**<br>**KAP 478 R**	For cloning downstream of *hupB*	**5' CGTATCTAGACGCCAAGAAGGCAACCGCTC 3'**<br>**5' TGTATCATGATCGAGGAGACCGGTCACCGCGC 3'**	This study
**KAP 485 R**<br>**KAP 486 F**	Hygromycin internal P1<br>Hygromycin internal P2 (Fig. S3A(ii))	**5' CTTGTGTCACAGCGGACCTC 3'**<br>**5' TGCTCGCCTTCACCTTCCTG 3'**	This study
**KAP 556 F**<br>**KAP 557 R**	For real time PCR of *sigA*	**5' CCATCCCGAAAAGGAAGACC 3'**<br>**5' TCGAGGTCTGGTTCAGCGTC 3'**	This study
**KAP 634 F**<br>**KAP 635 R**	For cloning *hlp*	**5′TCGAGCATATGATGAACAAAGCGGAGCTCATCGACGTACTCACAAC 3′**<br>**5′TCTGCAAGCTTTTACCTGCGGCCCTTCTTGGCCGGGGCCTTCTTGG 3′**	This study
ppsA* F**<br>ppsA* R**	For real time PCR of *ppsA*	**5' CACGACGTGCTGGCTAACGGCGAGG 3'**<br>**5' CCCGCAGACCCTCGGCCGGGGTCAG 3'**	This study
ppsC* F**<br>ppsC* R**	For real time PCR of *ppsC*	**5' AAATGCTTTCCAGGCTCGGTGTCGAG 3'**<br>**5' GGCGTAGACGTCCTTCTTGCCCAGT 3'**	This study
ppsE* F**<br>ppsE* R**	For real time PCR of *ppsE*	**5' CCGTTCATATCGCCGCCGCGCACGG 3'**<br>**5' GAGGGCCGCGCAACGCAGTGACCAT 3'**	This study
drrA* F**<br>drrA* R**	For real time PCR of *drrA*	**5' GAAAGAGGCGGGTGGGCACCTACT 3'**<br>**5' GAGATACTGCGTGGTCAACAACGTG 3'**	This study
katG* F**<br>katG* R**	For real time PCR of *katG*	**5' CCCTGAGATGACGGTGCTGGTAGG 3'**<br>**5' GGTCCACTTCACCTTGCCACTGC 3'**	This study

Table S4: MIC determination for SD1.

SD1 (μM)	Additional Growth (+/-)
0.00	+
0.39	+
0.78	+
1.56	+
3.12	+
6.25	+
12.5	+
25.0	+
50.0	+
100.0	-
200.0	-

Legend: Approximately 10^3 WT mycobacterial cells were used for the micro-dilution assay in 96 well U bottom plates and incubated for growth for 14 d. The lowest concentration where no pellet is visible is considered as the MIC for SD1.

Table S5: Checker board analysis of SD1 and INH combination.

INH (μM)	SD1 (μM)										
	200	100	50	25	12.5	6.25	3.12	1.56	0.78	0.39	0.0	Control
0.80	-	-	-	-	-	-	-	-	-	-	-	+
0.40	-	-	-	-	-	-	-	-	-	-	-	+
0.20	-	-	(-)	(-)	+	+	+	+	+	+	+	+
0.10	-	-	(-)	+	+	+	+	+	+	+	+	+
0.05	-	-	+	+	+	+	+	+	+	+	+	+
0.02	-	-	+	+	+	+	+	+	+	+	+	+
0.01	-	-	+	+	+	+	+	+	+	+	+	+
0.0	-	-	+	+	+	+	+	+	+	+	+	+

Legend: Approximately 10^3 WT cells were used for the checker board assay in 96 well U bottom plates and incubated for growth for 14 d. SD1 was diluted horizontally (in the indicated concentrations) and INH was diluted vertically (in the indicated concentrations). The fractional inhibitory concentration (FIC) and fractional inhibitory concentration index (FICI) were calculated for various concentrations of drug-combinations as in (Arora et al., 2020). + indicates growth; - indicates no growth; (-) enclosed in red encase indicates combinations of SD1 and INH where there is no growth; blue oval indicates MIC to INH (alone); and yellow oval indicates MIC to SD1 (alone).

Reference: Arora, G., Gagandeep, Behura, A., Gosain, T. P., Shaliwal, R. P., Kidwai, S., et al. (2020). NSC 18725, a Pyrazole Derivative Inhibits Growth of Intracellular Mycobacterium tuberculosis by Induction of Autophagy. *Frontiers in Microbiology* 10:3051. https://doi.org/10.3389/fmicb.2019.03051.
